# Functional electrical stimulation for major depressive disorder: Protocol for a pilot randomized controlled trial

**DOI:** 10.1371/journal.pone.0357381

**Published:** 2026-09-10

**Authors:** Fatemeh Gholamali Nezhad, Haochen Yu, Ilya Demchenko, Stephanie N. Iwasa, Stephanie DiNunzio, Rayna Ghosh, José Zariffa, Kei Masani, Wendy Lou, Benoit H. Mulsant, Hani E. Naguib, Milos R. Popovic, Venkat Bhat

**Affiliations:** 1 Interventional Psychiatry Program, St. Michael’s Hospital, Unity Health Toronto, Toronto, Ontario, Canada; 2 Institute of Medical Science, Temerty Faculty of Medicine, University of Toronto, Toronto, Ontario, Canada; 3 The KITE Research Institute, Toronto Rehabilitation Institute, University Health Network, Toronto, Ontario, Canada; 4 CRANIA, University Health Network and University of Toronto, Toronto, Ontario, Canada; 5 Institute of Biomedical Engineering, University of Toronto, Toronto, Ontario, Canada; 6 Rehabilitation Sciences Institute, University of Toronto, Toronto, Ontario, Canada; 7 Edward S. Rogers Sr. Department of Electrical and Computer Engineering, University of Toronto, Toronto, Ontario, Canada; 8 Lyndhurst Centre, KITE Research Institute–University Health Network, Toronto, Ontario, Canada; 9 Division of Biostatistics, Dalla Lana School of Public Health, University of Toronto, Toronto, Canada; 10 Department of Psychiatry, University of Toronto, Toronto, Ontario, Canada; 11 Centre for Addiction and Mental Health, Toronto, Ontario, Canada; 12 Department of Mechanical and Industrial Engineering, University of Toronto, Toronto, Ontario, Canada; 13 Li Ka Shing Knowledge Institute, St. Michael’s Hospital, Toronto, Ontario, Canada; Public Library of Science, UNITED STATES OF AMERICA

## Abstract

**Background:**

Conventional treatments for major depressive disorder (MDD) fail to benefit one-third of patients, underscoring the need for therapies with novel mechanisms of action. Bilateral functional electrical stimulation (FES) of the facial muscles uses electrical currents to activate the muscles involved in facial expression and may offer a novel “mind–body” intervention for MDD. Based on preliminary work demonstrating the feasibility of FES for MDD, this proposed study will assess the efficacy, safety, and tolerability of FES in MDD.

**Methods:**

This single-site, double-blind, randomized, sham-controlled pilot trial will enroll 60 participants to evaluate the therapeutic effects of 20 sessions of bilateral facial FES over four weeks. The study will assess the impact of FES on depressive symptoms, associated anxiety, quality of life, and sleep. Participant dropout and protocol adherence will be monitored to assess tolerability, while adverse events will be monitored to assess safety throughout the trial. Eligible participants will complete 20 on-site intervention visits and 6 online visits, including a screening visit, a baseline visit, and four post-stimulation follow-up visits.

**Discussion:**

The findings from this trial will guide the development of a multisite, large-scale randomized controlled trial and inform further refinement of FES treatment parameters for this population.

**Trial registration:**

This trial was registered at the National Library of Medicine, National Center for Biotechnology Information (ClinicalTrials.gov: NCT07629050. Date registered: 2026-06-08).

## Introduction

Major depressive disorder (MDD) is among the leading causes of disability worldwide, with an estimated global prevalence of 185.15 million cases and 274.80 million incident cases in 2019 [1]. While antidepressants are commonly recommended as first-line treatment for MDD, they have significant side effects and a therapeutic time lag [2–4]. Around 63% of MDD patients using second-generation antidepressants experienced at least one side effect and around 15% discontinued treatment as a result [2]. An alternative treatment modality for MDD patients who fail to respond to a course of antidepressants is neurostimulation, including hospital-based methods such as electroconvulsive therapy (ECT) and repetitive transcranial magnetic stimulation (rTMS) [5] along with home-based methods like transcranial direct current stimulation (tDCS) [6]. However, existing neurostimulation techniques are limited by variable efficacy and practical barriers related to treatment burden, invasiveness, and clinical implementation [7]. Thus, there is a need to investigate alternative neurostimulation modalities to treat MDD.

Functional electrical stimulation (FES) is a therapeutic technique that uses low-energy electrical impulses to activate neuromuscular units, which then stimulate skeletal muscles to contract and perform specific movements [8]. FES has been actively used in the context of neurorehabilitation and has been shown to be effective in restoring motor function following stroke and spinal cord injury through mechanisms of neuroplasticity [8–12]. Emerging research suggests that FES targeting muscles of facial expression could be a potentially effective modality for the treatment of MDD based on the facial feedback hypothesis, which postulates that facial expressions can influence and modulate an individual’s emotional state through proprioceptive and interoceptive feedback loops [13,14]. A non-Duchenne smile primarily involves activation of the zygomaticus major, whereas a Duchenne smile additionally recruits the orbicularis oculi and has been more consistently associated with positive emotional experience [13]. These smiles are mediated by different neural pathways. Experimental findings also suggest that combined activation of the zygomaticus major and orbicularis oculi may have a greater positive influence on emotional experience than activation of the zygomaticus major alone, providing a rationale for targeting both muscles with FES.

A neural feedback loop has been proposed in which facial muscle activity influences emotional processing through the amygdala, a key structure involved in emotion processing and generation [15]. Facial motor activity is conveyed to the amygdala, which integrates proprioceptive signals from the trigeminal nerve and interoceptive signals carried through the glossopharyngeal and vagus nerves [15]. By activating the facial muscles, FES may engage these pathways and contribute to neuroplastic changes involved in emotional regulation.

Zariffa et al. (2014) [14] conducted a pilot study exploring the mood-related effects of a single FES session targeting the Duchenne smile muscles in healthy individuals. Twelve participants receiving FES were compared with 12 controls. Based on the Positive and Negative Affect Schedule-X (PANAS-X), the findings suggested that FES may influence emotional states such as feeling “determined,” “daring,” “scared,” and “concentrating.” Building on this initial study, Kapadia et al. (2019) [16] conducted an open-label trial with MDD patients and reported significant improvements in depressive symptoms following a treatment course with FES, with participants showing an 8.1-point reduction on the 17-item Hamilton Depression Rating Scale (HAM-D-17) and a 14-point reduction on the Inventory of Depressive Symptomatology after 10 sessions of FES. Moreover, evidence from studies on Facial Neuromuscular Electrical Stimulation (fNMES), which directly activates facial muscles using computer-controlled electrical impulses, also supports an effect of facial muscle activation on emotional experience [17,18]. Participants reported more positive emotions after stronger stimulation of the zygomaticus major (smile muscle) compared to the depressor anguli oris (frown muscle) [18]. These studies provide promising evidence for the therapeutic potential of FES for MDD, setting the stage for designing a rigorous randomized sham-controlled trial (RCT) examining the efficacy of repetitive FES of facial muscles for MDD. This pilot RCT will assess the efficacy, safety, and tolerability of 20 sessions of FES in 60 participants diagnosed with MDD using a sham-controlled design.

### Objectives

The primary objective of this RCT is to evaluate the efficacy, tolerability, and safety of FES targeting the Duchenne muscles in participants with MDD. We hypothesize that participants receiving active FES will show greater reductions in the HAM-D-17 [19] total score than participants receiving sham FES with no significant differences in dropout rates, protocol adherence, or the incidence of adverse events (AEs) and serious adverse events (SAEs) between the two groups.

A secondary objective of this RCT is to evaluate the impact of FES on MDD response and remission rates, anxiety, quality of life, sleep quality, and the sustainability of these effects over four weeks. We hypothesize that participants receiving active FES will show significantly greater improvements across all these domains compared to those receiving sham FES.

A further objective of this RCT is to explore changes in facial expressivity during a semi-structured emotional recall interview using objective facial coding. We hypothesize that participants receiving active FES will demonstrate greater frequency, intensity, and duration of Duchenne smiles than those receiving sham FES during emotional recall interviews, reflecting enhanced emotional expressivity.

## Methods

### Study design and setting

**Fig 1:**
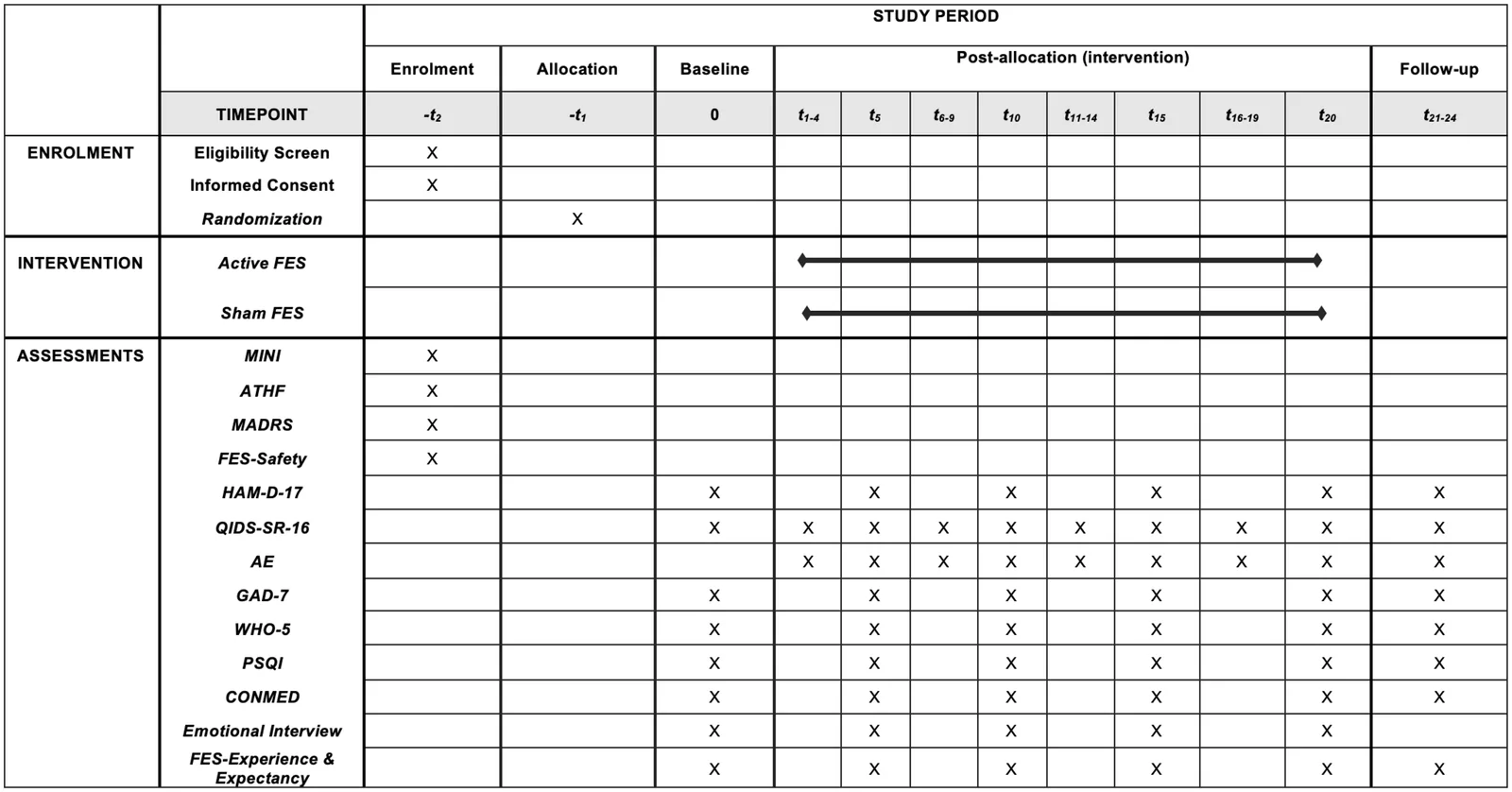
SPIRIT figure: schedule of enrollment, interventions, and assessments. Abbreviations: AE = Adverse Event, ATHF = Antidepressant Treatment History Form, CONMED = Concomitant Medication Record, FES = Functional Electrical Stimulation, GAD-7 = Generalized Anxiety Disorder-7, HAMD-17 = 17-Item Hamilton Depression Rating Scale, MADRS = Montgomery–Åsberg Depression Rating Scale, MINI = Mini-International Neuropsychiatric Interview, PSQI = Pittsburgh Sleep Quality Index, QIDS-SR-16 = 16-Item Quick Inventory of Depressive Symptomatology – Short Form, WHO-5 = World Health Organization-Five Well-Being Index.

**Fig 2:**
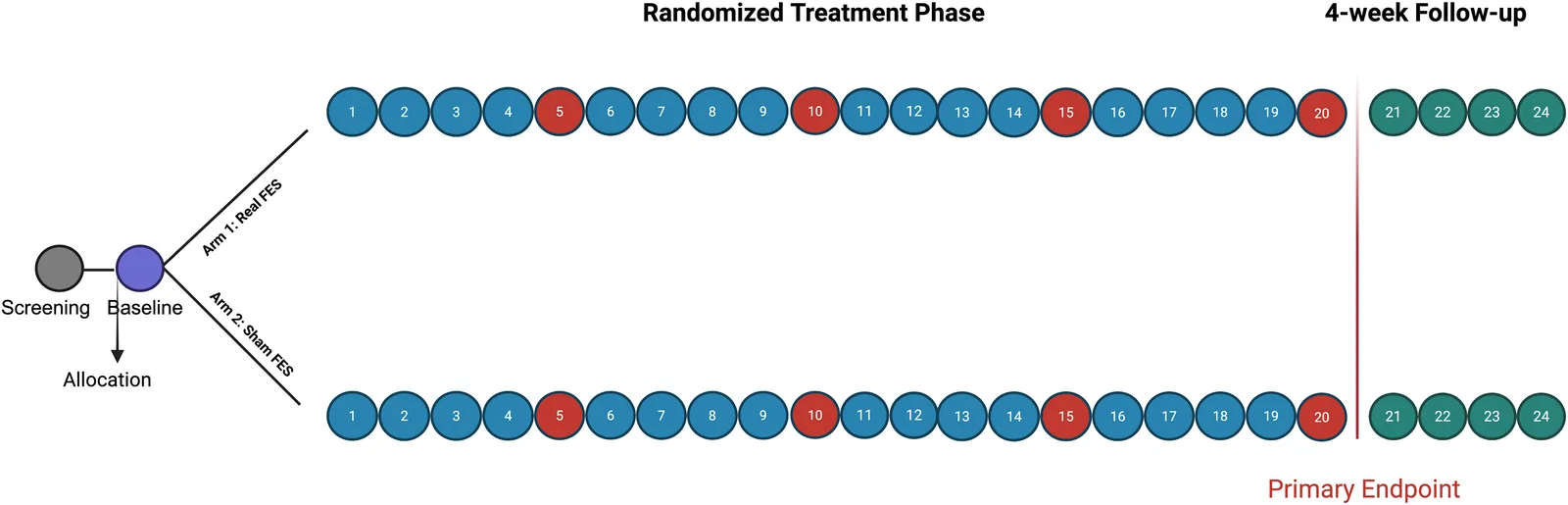
Study design. FES-Active and FES-Sham groups will complete a total of 26 visits. The 20 intervention visits will take place once per day over 20 days. Created in BioRender: RR29VRAKC1.

This study is a single-site, double-blind, pilot RCT conducted at St. Michael’s Hospital, Unity Health Toronto. The study design follows the SPIRIT guidelines (Fig 1) [20]. Eligible participants will be randomly assigned to receive either 20 sessions of active FES or sham. Both groups will complete a total of 26 visits (Fig 2). Six visits, including a screening visit, a baseline visit, and four follow-up visits, will be conducted remotely via phone or video conferencing using the Zoom platform. The 20 intervention visits will take place on-site, with participants attending one stimulation session per day over 20 consecutive working days. A trained staff member will directly monitor stimulation sessions. AEs and depressive symptoms will be monitored daily. All baseline assessments, except the emotional recall interview, will be repeated at the end of each intervention week and at each follow-up visit. The emotional recall interview will be conducted at baseline and at the end of each intervention week only. The SPIRIT Checklist is available in S1 File. The study protocol (Version 1, dated 10 March 2026; approved by the St. Michael’s Hospital Research Ethics Board, REB No. 24–155) is available as S2 File.

### Eligibility criteria

Recruitment will take place through referrals to the Interventional Psychiatry Program (IPP) at St. Michael’s Hospital, Unity Health Toronto, as well as through approved advertisements and flyers. Sixty participants with unipolar, non-psychotic MDD, as determined by the Mini-International Neuropsychiatric Interview (MINI) [21], will be recruited. Eligible participants will be males and females aged 18–70 years. They must have a Montgomery–Åsberg Depression Rating Scale (MADRS) [22] score of ≥7 and no more than two failed treatment trials during the current major depressive episode. Eligible participants will be required to maintain a stable regimen of medication and psychotherapy for at least four weeks before the study, and have no plan to change them throughout the intervention period, and during the follow-up, as reported in the Antidepressant Treatment History Form (ATHF) [23].

Individuals will be excluded if they have received ECT, magnetic seizure therapy, or intravenous ketamine during the current depressive episode, or rTMS within the previous 28 days. Additional exclusion criteria encompass a diagnosis of fibromyalgia, a history of epilepsy or seizures, oral metallic orthopedic implants, or damaged or dysfunctional facial nerves. Psychiatric comorbidities warranting exclusion consist of past or present symptoms of mania, hypomania, mixed episodes, psychotic disorders, obsessive-compulsive disorder, post-traumatic stress disorder, active substance use or dependence (excluding nicotine and caffeine), neurodegenerative disorders, or dementia. Participants with current suicidal intent or plan (MADRS item 10 score ≥ 4) or inability to generate the Duchenne smile with FES will also be excluded. Additionally, individuals who do not speak English will be excluded from this RCT to ensure clear comprehension of study information, facilitate communication for addressing participant questions, and obtain informed consent effectively.

### Sample size

The sample size calculation is based on a between-group comparison of the mean change in HAM-D-17 scores from baseline to day 20. Analysis of covariance (ANCOVA) will be used, with the change in HAM-D-17 score from baseline to day 20 as the outcome and baseline HAM-D-17 score and sex as covariates. In our previous pilot study [16], involving 10 participants with moderate to severe MDD who received three FES sessions per week (ranging from a minimum of 10 to a maximum of 40 total sessions), FES demonstrated a moderate to large effect size (d = 0.6–1.2) in MDD. All 10 participants completed the required 10 sessions, and 5 participants completed 40 sessions [16]. Our RCT will use 20 sessions of FES, which was well tolerated in research on rTMS for MDD and FES for stroke and spinal cord injury rehabilitation.

A total of 60 participants (30 in the active arm and 30 in the sham arm) is planned to detect a clinically meaningful between-group difference in HAM-D-17 scores, assuming a moderate effect size of 0.5 (Cohen’s d), a two-tailed significance level of 0.05, and 80% power. Accounting for a 15% attrition rate, approximately 51 participants are expected to complete the study, exceeding the recommended minimum sample size (24–50 participants) needed to generate reliable estimates of the standard deviation and correlation of HAM-D-17 scores for future sample size calculations in a definitive trial. Furthermore, a sample size of 60 will allow the anticipated 15% dropout rate to be estimated with an approximate 95% confidence interval margin of error of ±8.4. As the first proof-of-concept RCT of its kind, this trial is designed primarily to detect a signal for the primary outcome, change in HAM-D-17 over time. We recognize that the proposed sample size may not provide high statistical power for some secondary and exploratory outcomes.

### Recruitment

Participants referred to the IPP will be contacted to receive an overview of the study, have their questions addressed, and provide verbal consent for participation. Interested participants will then complete a pre-screening survey via Research Electronic Data Capture (REDCap) to determine preliminary eligibility. Individuals responding to social media advertisements or flyers will be directed to REDCap via the link provided in the advertisements to complete the same pre-screening survey. For those who meet initial eligibility criteria, a screening visit over the phone will be scheduled to assess full eligibility. The screening visit will include an eligibility review, collection of demographic data, medical history, confirmation of MDD diagnosis, and completion of an FES safety questionnaire. Verbal informed consent will be obtained before screening, and written informed consent will be obtained on the first day of on-site intervention sessions.

While there are no known risks of FES stimulation to ova or fetuses during pregnancy, the possibility of unknown risks cannot be entirely ruled out. Thus, female participants will be asked to confirm they are not pregnant or planning a pregnancy during the study. Upon meeting all requirements, a baseline visit will be scheduled.

### Randomization and masking

Participants will be randomly assigned in a 1:1 ratio to receive either active FES or sham using a randomization schedule generated through a secure REDCap module. Block randomization, stratified by sex, will account for potential differences in response and tolerability. Personnel conducting outcome assessments and other blinded study procedures will not have access to the fixed block size or treatment assignments. Randomization will be conducted by the intervention staff, who will have access to group assignments as required to deliver and monitor the stimulation sessions. In an emergency, the study doctor may be informed of the participant’s group assignment if this information is necessary for clinical management.

Moreover, to preserve blinding, sensory and non-Duchenne-patterned sham stimulation will be utilized. Participants will be instructed to avoid interaction with one another throughout the study. Blinding effectiveness will be assessed after each session by asking participants and raters to indicate whether they believe the participant received active or sham FES.

### Intervention

**Fig 3:**
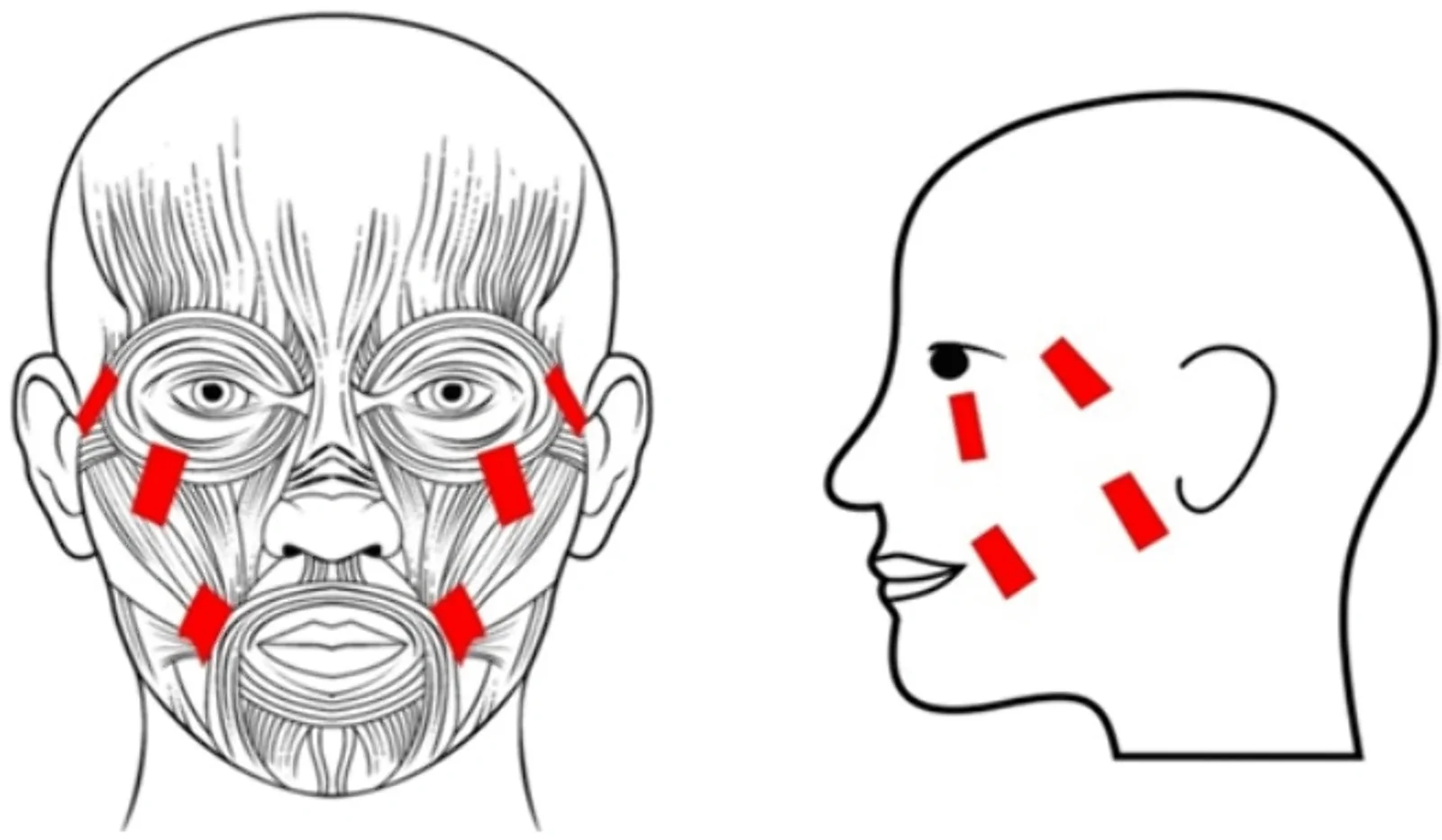
Surface electrode placement. FES of bilateral zygomaticus major and orbicularis oculi muscles.

Participants will receive transcutaneous FES targeting the bilateral zygomaticus major and orbicularis oculi muscles with self-adhesive surface electrodes placed on all four muscles (Fig 3). Each FES session, whether active or sham, will last for 60 minutes, comprising 15 minutes of preparation and 45 minutes of stimulation. FES will be administered using the Twin Stim® Plus Digital TENS/EMS FES device (Health Canada License name: TWIN STIM SERIES), providing 300 μs charge-balanced biphasic pulses at 40 Hz. During the intervention sessions, participants will watch a comedy video designed to elicit genuine Duchenne smiles while being instructed to intentionally produce a Duchenne smile at least once per minute. All intervention visits will be video recorded.

For the active group, a synchronized stimulation of four muscles (i.e., the bilateral zygomaticus major and bilateral orbicularis oculi) will be delivered. The current amplitude will range from 8 to 15 mA, which is sufficient to induce contractions in the desired target muscles, with alternating 15-second periods of stimulation and rest. These parameters align with previous FES studies for muscle activation [24]. If participants experience discomfort, the stimulation intensity may be adjusted within permitted parameters, and all adjustments will be documented.

Participants in the sham arm will receive FES with identical parameters, except that the stimulation will be limited to sensory levels (1–8 mA) and will follow a random circulating pattern rather than a Duchenne pattern. Although sham stimulation may cause some muscle contractions, it does not affect the study’s primary focus on examining the effects of Duchenne-pattern stimulation on mood.

### Outcome measures and data collection

The primary outcomes include changes in depressive symptoms, measured by the HAM-D-17, a validated, semi-structured, clinician-administered interview for assessing the presence and severity of depression. The other primary outcome will assess tolerability and safety based on dropout, data completion, protocol adherence rates, and the frequency and nature of AEs and SAEs. Secondary outcomes include MDD response and remission rates, anxiety, quality of life, and sleep quality. These outcomes will be measured using the 16-Item Quick Inventory of Depressive Symptomatology – Short Form (QIDS-SR-16) [25], Generalized Anxiety Disorder-7 (GAD-7) [26], World Health Organization-5 Well-Being Index (WHO-5) [27], and Pittsburgh Sleep Quality Index (PSQI) [28]. Additional secondary outcomes include response (50% decrease in total HAM-D-17 score) and remission (HAM-D-17 < 7) rates and sustainability of these outcomes for up to 28 days post-stimulation. In addition, facial expressivity during video-recorded emotional recall interviews will be assessed as an exploratory outcome.

An independent assessor will administer the HAM-D-17 and complete the Concomitant Medication Record (CONMED) at baseline, at the end of each week during the randomized intervention period, and at each follow-up visit. Participants will also be queried daily about their experiences and any AEs or SAEs they may encounter. To monitor participant burden, an FES Experience assessment will be conducted at the end of each treatment week through REDCap. All secondary outcomes will be assessed at baseline, at the end of each week during the randomized intervention period, and during follow-up visits using REDCap. Emotional recall interview will be administered at baseline and at the end of each week during the intervention period.

This study will also collect necessary personal health information, including the participant’s name, date of birth, medical records (new or existing) related to psychiatric or medical conditions, current and past medications, surgical implants, and any illnesses or psychiatric procedures that could affect study participation. This information will be obtained from the participant, their physician, or their medical records.

### Data management, confidentiality, and monitoring

REDCap will be used as the primary platform for data collection in this study. Participants will complete self-report questionnaires through REDCap links sent on a daily and weekly basis. Clinician-administered assessments (i.e., MINI, HAM-D-17, and MADRS) will be conducted by trained study staff. All data will be securely stored on the REDCap server and linked with a unique identifier for analysis. The screening survey will collect personally identifiable information, whereas the REDCap forms used for outcome assessments will use only a unique study identification number. Personally identifiable information will be kept confidential in accordance with applicable privacy legislation, including Ontario’s Personal Health Information Protection Act (PHIPA).

Video recordings, which contain identifiable facial and voice information, will be stored on a secure, password-protected hard drive within the Unity Health Toronto environment, accessed only by authorized study personnel, and processed locally using OpenFace; only coded numerical facial-movement data linked to study identification numbers will be used for analysis, and no recordings will be uploaded to external websites, cloud services, or third-party servers.

To assess data quality, a second reviewer will verify the data from a randomly selected 10% of participants. Screening data from individuals who are found to be ineligible will be retained unless they request their removal, provided that the data have not already been anonymized or incorporated into aggregate analyses. All study records will be stored securely for seven years in accordance with institutional research ethics requirements.

### Safety and adverse events monitoring

Participant safety during all FES sessions will be overseen by trained investigators, with a licensed physician available at every visit. All AEs will be systematically recorded, including their severity, causality, and outcomes. AEs will be assessed daily: 1) the severity of each AE will be graded as mild, moderate, or severe, 2) causality will be categorized as probable, possible, unlikely, not related, or unclassified. SAEs are defined as events leading to significant disability, death, congenital defects, hospitalization, or requiring intervention to prevent such outcomes. Any SAE will be reviewed immediately and reported in accordance with ethical guidelines.

The outcome of each AE will be recorded as recovered/resolved, recovering/resolving, not recovered/not resolved, recovered/resolved with sequelae, fatal, or unknown. The study physician will promptly evaluate any unexpected events requiring urgent attention. In the event of an AE, medical care will be facilitated for the participant, and follow-up will be conducted by the Principal Investigator (PI). The PI will review and initial the AE log within 72 hours of its completion. All AEs, regardless of their relationship to FES, will be documented, followed until adequate resolution, and graded for severity and causality.

### Patient retention and adherence

To develop professional relationships with participants, a specific research team member will be assigned to each participant for consistency across all visits. Participants will receive $30 for each on-site visit. Participants are allowed to miss up to two intervention visits during the intervention period. Missed visits will be recorded as non-adherent in the database and rescheduled immediately after the planned intervention period so that participants can complete all treatment sessions without substantial delay.

### Data analysis plan

Baseline demographic and clinical characteristics will be summarized for each treatment group. The primary analyses will be conducted on a modified intention-to-treat (ITT) sample, including all randomized participants who received at least one FES session. The efficacy of FES will be evaluated using ANCOVA to compare changes in HAM-D-17 scores from baseline to the final FES session between the active and sham groups, adjusting for baseline HAM-D-17 score and sex. Correlations between baseline and follow-up HAM-D-17 scores will be analyzed at the participant level. Similar analyses will be conducted for secondary clinical outcomes. Moreover, FES tolerability will be evaluated by summarizing overall dropout rates as counts and percentages. For dichotomous outcomes like remission, response, and AEs, between-group differences will be analyzed using odds ratios with 95% confidence intervals. Blinding effectiveness will be assessed by calculating Bang’s blinding index for rater and participants. Analyses stratified by sex and age will also be performed to explore potential subgroup differences.

Additionally, automated facial behavior analysis software (OpenFace) will be used to quantify Duchenne smile metrics derived from facial Action Units (AUs), including frequency, duration, and average activation intensity. Duchenne smiles will be identified based on the concurrent activation of AU12 (lip corner puller) and AU6 (cheek raiser). Descriptive statistics and group comparisons will be performed to examine differences in genuine smiling between the FES and sham groups. OpenFace is an open-source facial behavior analysis toolkit developed by researchers at the University of Cambridge and Carnegie Mellon University. Using computer vision and machine learning algorithms, it detects facial landmarks (e.g., eye, mouth, and eyebrow positions), estimates head pose, and quantifies facial muscle activity through the Facial Action Coding System (FACS) by identifying the presence and intensity of facial AUs. Participant videos will be processed using OpenFace to extract facial landmark coordinates and AU measurements. The output will consist of CSV files containing numerical facial behavioral data identified only by participants’ study IDs. These de-identified data will subsequently be used for statistical analysis.

### Participant withdrawal and study termination

Participants may withdraw their consent and discontinue the study at any time without penalty. Withdrawal may also be initiated by the participant’s physician or the investigator if continued participation is deemed to pose a significant risk. Discontinuation criteria include a worsening of depression (defined as a more than 25% increase in QIDS-SR-16 score compared to baseline across two consecutive assessments), developing active suicidal intent, attempting suicide, non-adherence to study procedures, or missing more than two FES sessions. Participants meeting exclusion criteria during the trial will also be withdrawn. All early withdrawals will be documented, and participants will be asked to provide feedback on their FES experience and undergo any recommended medical evaluations. If study termination or early withdrawal is due to an AE or SAE, the research team will assist the participant in obtaining appropriate medical care, and the PI will conduct follow-up as required.

The study will be paused if SAEs related to FES are identified. Premature termination may occur if significant risks to participants, protocol non-adherence, insufficient evaluable data, or device modifications are identified. In such cases, the research ethics board will be informed.

### Study status and timeline

Recruitment began on June 20, 2026, and is currently ongoing. Participant recruitment is expected to be completed by June 2028, data collection by December 2028, and the primary study results are expected by June 2029.

### Success criteria

The success of this study will be assessed quantitatively based on the achievement of its primary and secondary objectives. Success will be evaluated across three domains: efficacy, tolerability, and safety, based on changes in HAM-D-17 scores, participant retention and protocol adherence, and the frequency and nature of AEs and SAEs. Specifically, success will be indicated by a clinically meaningful reduction in HAM-D-17 scores, defined as a reduction of ≥50% from baseline, in the active FES group. Additionally, a statistically significant difference in HAM-D-17 score changes between the active and sham groups will be required. Successful tolerability will be demonstrated by a dropout rate of ≤15% and high adherence to the treatment protocol, with at least 85% of participants completing all scheduled FES sessions.

For safety outcomes, the number, nature, and duration of AEs will be evaluated in both active and sham groups, and no significant differences between groups in AE frequency or severity will indicate successful safety outcomes. Additionally, the sustainability of observed treatment effects will be assessed by analyzing HAM-D-17 response and remission rates, as well as changes in secondary outcomes, including anxiety (GAD-7), quality of life (WHO-5), and sleep quality (PSQI) over the four-week follow-up period. Sustained improvements across these domains in the active group, without significant increases in adverse effects, will further validate the success of the study.

If these success criteria are met, the findings will provide a robust foundation for designing a large-scale, multisite RCT to further evaluate FES for MDD.

## Discussion

To our knowledge, this study is the first RCT to evaluate the efficacy of FES in MDD. This study represents a significant advancement in exploring FES as a therapeutic modality for MDD. It will compare the outcomes of 20 sessions of active versus sham FES, specifically targeting the Duchenne smile muscles. The primary outcomes include efficacy assessed through changes in HAM-D-17 scores, while tolerability and safety will be evaluated through compliance and dropout rates. Secondary outcomes, including HAM-D-17–defined response and remission, anxiety, sleep quality, and quality of life, will also be assessed for up to four weeks after treatment.

Recent work has highlighted fNMES as a method for investigating facial feedback and the growing overlap between neurorehabilitation and psychiatry [29], supporting the investigation of FES as a treatment for MDD. The rationale for this approach is based on the facial feedback hypothesis, which suggests that facial expressions can modulate emotional states through interoceptive and proprioceptive feedback loops [13,14]. By stimulating the zygomaticus major and orbicularis oculi muscles, which are involved in the Duchenne smile, FES may engage these feedback mechanisms and potentially induce neuroplastic changes in neural circuits involved in emotional regulation.

Given the substantial burden of MDD and the limitations of current antidepressant treatments, interest has grown in alternative or adjunctive approaches, including electrical stimulation of facial muscles to modulate mood and emotion and potentially reduce depressive symptoms [14,16,18,30]. Stimulation of the zygomaticus major (smiling muscle) led to more positive emotional reports compared to stimulation of the depressor anguli oris (frowning muscle), even after controlling for discomfort [18]. Pilot studies targeting the muscles involved in Duchenne smiling in healthy individuals and participants with MDD have provided preliminary support for this approach and suggest that FES is well tolerated, with reported AEs limited to mild, transient discomfort [14,16]. This suggests that the emotional effects arise from muscle activation itself rather than sensory stimulation.

Efthimiou et al. (2024) [30] provided further evidence for the facial feedback hypothesis by showing that fNMES of the zygomaticus major increased the likelihood that ambiguous faces expressions would be perceived as happy. The stimulation also affected early (P1, N170) and late (LPP) neural components. These findings indicate that electrical stimulation of facial muscles influences not only emotion recognition but also its underlying neural mechanisms, providing additional evidence for the role of FES in emotion regulation.

Building on this foundation, the current RCT aims to provide rigorous evidence on the efficacy, tolerability, and safety of FES in a larger sample of patients with MDD. If successful, this study will inform the design of large-scale, multisite RCTs to validate these findings and refine the treatment protocol. Additionally, FES holds promise as a self-administered, home-based neurostimulation treatment, offering an accessible and scalable therapy for individuals with MDD.

## Supporting information

S1 File SPIRIT checklist. (DOCX)

S2 File Study protocol. (DOCX)
