# Supplementary material for: Functional electrical stimulation for major depressive disorder: Protocol for a pilot randomized controlled trial
Source: PLoS One. 2026 Sep 10;21(9):e0357381. doi: 10.1371/journal.pone.0357381 (PMC13561349; doi:10.1371/journal.pone.0357381)
Supplement: Supplementary file 2 [file pone.0357381.s002.docx]

**–	STUDY PROTOCOL	–**

**Study Title****:** **Functional Electrical Stimulation for Major Depressive Disorder:** **A Pilot Randomized Control Trial**

**Study Code:**

**Protocol No.:**

**Amendment No.:**

**Study Principal Investigator:**

Venkat Bhat, MD MSc FRCPC DABPN

Director, Interventional Psychiatry Program, St. Michael's Hospital, Unity Health Toronto

Assistant Professor, Department of Psychiatry, University of Toronto

193 Yonge Street 6-013, Toronto, ON M5B 1M8, Canada

Tel : 416-864-6060 x76404	Fax : 416-864-5996	Email : [venkat.bhat@utoronto.ca](mailto:venkat.bhat@utoronto.ca)

**Co-Principal Investigators:**

Milos R Popovic, PhD

Director, KITE Research Institute, Toronto Rehabilitation Institute, University Health Network

Professor, Institute of Biomedical Engineering, University of Toronto 
550 University Ave., Room 12-157, Toronto, ON, M5G 2A2, Canada
Tel: 416-597-3422 x7628 [Email: milos.popovic@uhn.ca](mailto:Email:%20milos.popovic@uhn.ca)

**Co-Investigators:**

Wendy Lou, PhD

Professor, Division Head of Biostatistics, Dalla Lana School of Public Health, University of Toronto

Health Sciences Building, 155 College Street, Toronto, ON M5T 3M7;

Email: [wendy.lou@utoronto.ca](mailto:wendy.lou@utoronto.ca)

Benoit Mulsant, MD, MS, FRCPC

Professor, Labatt Family Chair of the Department of Psychiatry, University of Toronto

5 King’s College Rd., Toronto, ON, M5S 3G8 Canada

Email: benoit.mulsant@utoronto.ca

José Zariffa, PhD

Senior Scientist, KITE Research Institute – University Health Network

Associate Professor, Institute of Biomedical Engineering, University of Toronto

Email: [jose.zariffa@utoronto.ca](mailto:jose.zariffa@utoronto.ca)

Kei Masani, PhD

Associate Professor, Motion & Adaptation Science Laboratory, Lyndhurst Centre, Toronto Rehabilitation Institute

520 Sutherland Drive, Toronto, Ontario M4G 3V9 Canada

Email: [k.masani@utoronto.ca](mailto:k.masani@utoronto.ca)

**Study Funder:** Canadian Institutes of Health Research (CIHR)

**REVISION HISTORY**

| Version | Date | Amendment Text | Description |
| --- | --- | --- | --- |
| 1 | 03-Oct-2025 | The study design has been revised to one session per day throughout the protocol. | This change was made because (1) our experience with another ongoing FES project indicates that requiring participants to remain onsite for more than three hours per day is not feasible and substantially reduces recruitment, and (2) we have added tasks to each FES session that are likely to increase fatigue (including muscle fatigue), which could reduce tolerability if we retained the twice-daily protocol. |
| 1 | 03-Oct-2025 | Adding another secondary aim: To assess changes in facial expressions during semi-structured emotional recall interviews, using objective facial coding | To incorporate objective measures capturing changes in positive-affect expressions, particularly the Duchenne smile over time.  Data management and privacy sections are modified accordingly. |
| 1 | 03-Oct-2025 | Recruitment strategies now include flyers at SMH, Instagram ads, and advertising in public spaces (e.g., TTC). | Because the IPP population is largely treatment-resistant, enrolling 60 participants within the planned timeframe is unlikely; accordingly, we have expanded community outreach and will monitor accrual monthly, with contingencies to adjust timelines as needed. |
| 1 | 03-Oct-2025 | We are replacing the automated audios with short videos featuring positive/comedic content, clear verbal instructions on producing a Duchenne smile, and a participant button-press to confirm successful smile generation. | The prior audios were intended to cue smiling to facilitate contractions; however, pilot testing showed they did not reliably elicit the desired contraction pattern, and continuous audio for ~45 minutes was reported as annoying by some people who tested that. |
|  |  | All FES visits will be video recorded. | All FES visits will be video recorded to verify that the intended smile pattern is elicited, to confirm that each button press corresponds to an actual smile response, and to facilitate more accurate analysis. |
|  |  | Corrections to data management section | As we are using REDCap for data collection, there is no need for storing data in Microsoft Access. |
|  |  | 300 μs long charge-balanced biphasic pulses will be used for FES. | To be consistent with the other study on the feasibility of take-home FES. |

**Table of Contents**

[List of Abbreviations	8](#_Toc168398602)

[PROTOCOL SIGNATURES	10](#_Toc168398603)

[1. Trial Summary	12](#_Toc168398604)

[2. Introduction	16](#_Toc168398605)

[3. Background and Rationale	17](#_Toc168398606)

[4. Study Objectives	20](#_Toc168398607)

[5. Subject Selection and Recruitment	20](#_Toc168398608)

[5.1. Eligibility Criteria	21](#_Toc168398609)

[Inclusion Criteria	21](#_Toc168398610)

[Exclusion Criteria	21](#_Toc168398611)

[5.2. Concomitant Medications (CONMED)	22](#_Toc168398612)

[5.3. Method of Recruitment	22](#_Toc168398613)

[5.4. Retention Strategy, Costs, and Reimbursements	22](#_Toc168398614)

[6. Study Design	23](#_Toc168398615)

[6.1. Research design and methods	23](#_Toc168398616)

[6.2. Randomization and Blinding	27](#_Toc168398617)

[6.3. Study Schedule	27](#_Toc168398618)

[•	Inclusion/exclusion review	28](#_Toc168398619)

[•	Demographic review	28](#_Toc168398620)

[•	Review of medical, psychiatric, and medication history, including review and confirmation of diagnosis	28](#_Toc168398621)

[•	FES safety questionnaire	29](#_Toc168398622)

[6.4. Clinical Scales and Self-Report Measures	31](#_Toc168398623)

[6.5. REDCap	32](#_Toc168398624)

[7. Trial Intervention	33](#_Toc168398625)

[7.1. 2FES Active (motor and sensory stimulation):	33](#_Toc168398626)

[7.2. FES Sham (Sensory & non-patterned stimulation):	34](#_Toc168398627)

[7.3. Study Investigational Product	35](#_Toc168398628)

[7.3.1. Device Overview	35](#_Toc168398629)

[7.3.2. Operation of the device	35](#_Toc168398630)

[7.3.3. Device Safety Features	35](#_Toc168398631)

[8. Outcome Measures	35](#_Toc168398632)

[8.1. Primary Outcome Measures	35](#_Toc168398633)

[8.2. Secondary Outcome Measures	35](#_Toc168398634)

[9. Potential Risks and Benefits	36](#_Toc168398635)

[9.1. Potential Benefits	36](#_Toc168398636)

[9.2. Potential Risks and Analysis of Risk/Benefit Ratio	36](#_Toc168398637)

[10. Data and Safety Monitoring	37](#_Toc168398638)

[10.1. Quality Assurance	37](#_Toc168398639)

[10.3. Safety Oversight	37](#_Toc168398640)

[10.4. Early Withdrawal	37](#_Toc168398641)

[10.5. Study Termination	38](#_Toc168398642)

[11. Safety and Reporting	39](#_Toc168398643)

[11.1. Safety Parameters	39](#_Toc168398644)

[11.1.1. Adverse Event Monitoring	39](#_Toc168398645)

[11.1.2. Data Safety Monitoring Board (DSMB)	39](#_Toc168398646)

[Given that this is a feasibility trial with a small sample size, no Data Safety Monitoring Committee will be formed.	39](#_Toc168398647)

[11.1.3. General Safety Plan	39](#_Toc168398648)

[11.2. Adverse Events (AEs)	40](#_Toc168398649)

[11.2.1. Adverse Event definition	40](#_Toc168398650)

[11.2.2. Collection of AEs	40](#_Toc168398651)

[11.2.3. Severity of AEs	41](#_Toc168398652)

[11.2.4. Causality of AEs	41](#_Toc168398653)

[11.2.5. Outcome of AEs	42](#_Toc168398654)

[11.3. Serious Adverse Events (SAEs)	42](#_Toc168398655)

[11.4. Unexpected AEs and SAEs	43](#_Toc168398656)

[11.5. Reporting Procedures	43](#_Toc168398657)

[11.6. AEs and SAEs Follow-up	44](#_Toc168398658)

[11.7. Auditing	44](#_Toc168398659)

[12. Data Collection and Data Management	44](#_Toc168398660)

[12.1. Clinical Data Capture and Management	44](#_Toc168398661)

[Study data will be collected via case report forms (CRF). The study data will be entered into an	44](#_Toc168398662)

[encrypted and password protected Microsoft® Access database (Microsoft Corp., Redmond, WA,	44](#_Toc168398663)

[USA) by designated research personnel. Appropriate range and missing data filters will be used	45](#_Toc168398664)

[to address data quality. Data accuracy will be assessed by randomly selecting 10% of individuals	45](#_Toc168398665)

[who will have their data confirmed by a second reviewer.	45](#_Toc168398666)

[All records pertaining to the trial will be retained and stored for a period of 7 years as per Unity Health requirements.	45](#_Toc168398667)

[12.2. Privacy, Security, and Data Confidentiality	45](#_Toc168398668)

[12.3. Source Documents	45](#_Toc168398669)

[Unity Health REB Documents	46](#_Toc168398670)

[Informed Consent	46](#_Toc168398671)

[12.4. Data Management Responsibilities	46](#_Toc168398672)

[12.5. Protocol Deviations	46](#_Toc168398673)

[13. Ethical Considerations	46](#_Toc168398674)

[13.1. Ethical Standard	47](#_Toc168398675)

[13.2. Research Ethics Boards (REBs)	47](#_Toc168398676)

[13.3. Informed Consent Process	47](#_Toc168398677)

[13.4. Exclusion of women, minorities, and children (special populations)	48](#_Toc168398678)

[13.5. Confidentiality	48](#_Toc168398679)

[13.6. Study Discontinuation	49](#_Toc168398680)

[14. Data Analysis	49](#_Toc168398681)

[14.1. Sample Size Considerations	49](#_Toc168398682)

[14.2. Outcomes Analysis	49](#_Toc168398683)

[15. Publication Policy and Dissemination	50](#_Toc168398684)

[15.1. Clinical Trial Registration	50](#_Toc168398685)

[15.2. Declaration of Interests	50](#_Toc168398686)

[15.3. Dissemination Policy	51](#_Toc168398687)

[15.4. Authorship	51](#_Toc168398688)

[16. References	51](#_Toc168398689)

**16. References………………………………………………………………………………………….47**

# List of Abbreviations

| AE | Adverse Event |
| --- | --- |
| AEL | Adverse Event Log |
| ATHF | Antidepressant Treatment History Form |
| CAD | Computer-Aided Design |
| CONMED | Concomitant Medications |
| CONSORT | Consolidated Standards of Reporting Trials |
| CRF | Case Report Form |
| DSM-IV | Diagnostic and Statistical Manual of Mental Disorders, 4th Ed. |
| DSM-5 | Diagnostic and Statistical Manual of Mental Disorders, 5th Ed. |
| DSMB | Data Safety Monitoring Board |
| ECT | Electroconvulsive therapy |
| EMS | Electrical Muscle Stimulation |
| FES | Functional Electrical Stimulation |
| GAD-7 | Generalized Anxiety Disorder-7 Scale |
| GCP | Good Clinical Practice |
| HAM-D-17 | Hamilton Depression Rating Scale – 17-Item |
| ICF | Informed consent form |
| IMP | Investigational Medicinal Product |
| IV | Intravenous |
| KT | Knowledge Translation |
| MADRS | Montgomery-Åsberg Depression Rating Scale |
| MDD | Major Depressive Disorder |
| MINI | Mini-international neuropsychiatric interview |
| PSQI | Pittsburgh Sleep Quality Index |
| PT | Physiotherapist |
| QIDS-SR-16 | 16-Item Quick Inventory of Depressive Symptomatology |
| RC | Research Coordinator |
| RCT | Randomized controlled trial |
| REB | Research Ethics Board |
| rTMS | Repetitive transcranial magnetic stimulation |
| SAE | Serious Adverse Event |
| tACS | Transcranial alternating current stimulation |
| TCPS2 | Tri-Council Policy Statement |
| tDCS | Transcranial direct current stimulation |
| TENS | Transcutaneous Electrical Nerve Stimulation |
| WHO-5 | World Health Organization-5 Well-Being Index |
|  |  |

# PROTOCOL SIGNATURES

**Title: Functional Electrical Stimulation for Major Depressive Disorder: A Pilot Randomized Control Trial**

This study is intended to be conducted in compliance with the protocol, the International Council of Harmonization – Good Clinical Practice (ICH-GCP) E6, and applicable regulatory requirements.

**Principal Investigator**

Venkat Bhat, MD MSc FRCPC DABPN

Director, Interventional Psychiatry Program, St. Michael's Hospital, Unity Health Toronto

Assistant Professor, Department of Psychiatry, University of Toronto

| Signature |  | Date |
| --- | --- | --- |

**Co-Principal Investigator**

Milos R Popovic, PhD

Director, KITE Research Institute, Toronto Rehabilitation Institute, University Health Network

Professor, Institute of Biomedical Engineering, University of Toronto

| Signature |  | Date |
| --- | --- | --- |

**Co-Investigators**

Hani Naguib, PhD

Professor and Director, University of Toronto

| Signature |  | Date |
| --- | --- | --- |

Wendy Lou, PhD

Professor, Division Head of Biostatistics, Dalla Lana School of Public Health, University of Toronto

| Signature |  | Date |
| --- | --- | --- |

Benoit Mulsant, MD, MS, FRCPC

Professor, Labatt Family Chair of the Department of Psychiatry, University of Toronto

| Signature |  | Date |
| --- | --- | --- |

José Zariffa, PhD

Associate Professor, Senior Scientist at KITE, University of Toronto

| Signature |  | Date |
| --- | --- | --- |

Kei Masani, PhD

Associate Professor, Motion & Adaptation Science Laboratory, Lyndhurst Centre, Toronto Rehabilitation Institute

| Signature |  | Date |
| --- | --- | --- |

# 1. Trial Summary

| Title | Functional Electrical Stimulation for Major Depressive Disorder: a pilot randomized control trial |
| --- | --- |
| Primary registry and trial identifying number | ClinicalTrials.gov  NCT07629050 |
| Sources of monetary or material support | Canadian Institutes of Health Research (CIHR) |
| Study officials/Investigators | Study Principal Investigator:  Venkat Bhat, MD MSc FRCPC DABPN  St. Michael's Hospital, Unity Health Toronto, Toronto, ON, Canada  [venkat.bhat@unityhealth.to](mailto:venkat.bhat@unityhealth.to)  Study Co-Principal:  Milos R Popovic, PhD  Professor, Institute of Biomedical Engineering, University of Toronto  [milos.popovic@uhn.ca](mailto:milos.popovic@uhn.ca)  Co-Investigators:  Wendy Lou, PhD  Professor, Division Head of Biostatistics, Dalla Lana School of Public Health, University of Toronto  [wendy.lou@utoronto.ca](mailto:wendy.lou@utoronto.ca)  Benoit Mulsant, MD, MS, FRCPC  Professor, Labatt Family Chair of the Department of Psychiatry, University of Toronto  [benoit.mulsant@utoronto.ca](mailto:benoit.mulsant@utoronto.ca)  José Zariffa, PhD  Associate Professor, Senior Scientist at KITE, University of Toronto  [jose.zariffa@utoronto.ca](mailto:jose.zariffa@utoronto.ca)  Kei Masani, PhD  Associate Professor, Motion & Adaptation Science Laboratory, Lyndhurst Centre, Toronto Rehabilitation Institute  [k.masani@utoronto.ca](mailto:k.masani@utoronto.ca) |
| Brief title | FES for Depression |
| Countries of recruitment | Canada |
| Condition(s) or focus of study | Major Depressive Disorder (MDD) |
| Interventions |  |
| FES | Intervention type: Procedure/Surgery  Intervention name: Functional Electrical Stimulation (FES)  Intervention description: 40 Hz FES stimulation, 45 mins per day for the duration of 4 weeks (20 sessions over 20 days, with one session per day). |
| Sham Stimulation | Intervention type: Procedure/Surgery  Intervention name: Sham Stimulation  Intervention description: Sham stimulation, 45 mins per day for the duration of 4 weeks (20 sessions over 20 days, with one session per day). |
| Eligibility criteria | Inclusion Criteria:   1. Meet the DSM-5 (67) criteria for unipolar MDD with a current major depressive episode (MDE) without psychotic features, with ≤ 2 failed treatment trials (non-treatment-resistant depression), as validated by MINI (done by trained research assistant). 2. No change in the medication regimen or other forms of treatments (e.g., psychotherapy) for at least 4 weeks (28 days) prior to beginning the study, during the 20-session treatment period, and the 4-week post-treatment observation period. This will be established through self-report, in combination with the Antidepressant Treatment History Form (ATHF) form filled out by the participant. 1. MDD diagnosis as confirmed by a Montgomery-Åsberg Depression Rating Scale (MADRS) total score of ≥ 7 3. Age group between 18 and 70 years of age.   Exclusion Criteria:   1. History of epilepsy or seizures. 2. Damage or dysfunction of facial nerves. 3. Metallic implants or metal braces near the potential sites of electrical stimulation and any type of implanted electronic device 4. Current fibromyalgia or currently receiving or have received repetitive transcranial magnetic stimulation (rTMS) within the last month (28 days) before screening. 5. History of treatment-resistant depression (TRD) with history of ECT, Magnetic Seizure Therapy, Intravenous Ketamine use in the past or failure of >2 antidepressant treatments of adequate duration and dose during the current episode. 6. Past or current symptoms of mania, hypomania, mixed episodes, psychotic disorders, active substance abuse, or dependence (excluding nicotine and caffeine) which will be confirmed on the MINI done by trained research assistant. 7. Current suicidal intent or plan as demonstrated by a score of ≥ 4 on MADRS item 10. 8. Unable to understand instructions in English. 9. Unable to produce “Duchenne marker” expression with FES, secondary to any type of neurological condition or previous botulinum toxin treatments of facial muscles. |
| Study design | Study type: Interventional trial  Allocation: Randomized  Intervention model: Parallel arm  Primary purpose: Efficacy  Phase: Pilot study |
| Masking | Double-blind |
| Date of enrollment | Immediately upon REB approval |
| Target sample size | 60 |
| Primary outcomes | Improvement in symptoms of depression (HAM-D-1) at the end of the 2-week treatment, response/remission rates, and sustainability of effects 4 weeks post-intervention.  The tolerability and safety outcomes of recruitment dropout, data completion, and protocol compliance rates, and the number and nature of adverse events and serious adverse events, to evaluate patient experience with the FES |
| Secondary outcomes | Improvements in self-reported symptoms of anxiety (GAD-7), quality of life (WHO-5), sleep (PSQI)  Changes in facial expressions during semi-structured emotional recall interviews, using objective facial coding |

# 2. Introduction

A substantial proportion of Canadians suffer from Major Depressive Disorder (MDD), with an annual prevalence of 4.8% and lifetime prevalence of 12.2%^4^. MDD is the leading cause of disability, and currently available treatments for MDD are associated with side effects and low adherence^5,6^. Hospital-based neurostimulation modalities (e.g., electroconvulsive therapy [ECT], repetitive transcranial magnetic stimulation [rTMS]) for MDD are difficult to access, are associated with high costs for delivery in-hospital for acute treatment of MDD, and maintenance treatments are often needed to sustain efficacy^6-8^. While there are few low-cost treatments with potential for home-based use (e.g., light therapy, transcranial direct current stimulation [tDCS], computer-based psychotherapy), their efficacy is modest^9-11^. Thus, there is a critical need for efficacious and well-tolerated neurostimulation treatments which could be self-administered at a frequency required to treat MDD, maintain response, and reduce relapse. Efficacious and well-tolerated neurostimulation treatments with potential for home-based use will offer an alternative treatment modality for Canadians suffering from MDD, with the potential to significantly lower the load on Canadian hospitals/healthcare systems offering in-hospital treatments.

A potential novel intervention for MDD is bilateral functional electrical stimulation (FES) of the facial muscles. FES involves a systematic and coordinated application of electrical current to excitable tissues. FES has been extensively researched for restoring motor function in people with neurological deficits from stroke and spinal cord injury^14^. Two pilot feasibility studies with FES have been conducted showing that FES modulates mood and affect – one among healthy participants^15^, followed by repetitive FES for MDD participants^17^. The FES stimulator is presented in **Figures 1**.

**Figure 1:** Twin Stim® Plus Digital TENS/EMS stimulator

Based on the existing FES literature for neurological deficits reporting efficacy after 20 sessions, with each session lasting 45 minutes^20^, as well as preliminary work demonstrating the feasibility of ten days of FES for MDD, the proposed interdisciplinary collaboration will perform a pilot randomized control trial for 60 participants with MDD in 20 session, over 2 years.

# 3. Background and Rationale

Pharmacotherapy remains the mainstay of MDD treatment^21^ and is associated with significant adverse effects^22^. Non-pharmacological interventions for MDD include psychotherapy and neurostimulation^23^, which are often difficult to access. Neurostimulation treatments (e.g., ECT & rTMS) are hospital-based and not currently adaptable for home use; modalities such as tDCS and tACS have demonstrated limited efficacy^2,24^. Thus, there is an immediate need to explore treatments with alternate mechanisms of action, including the role of interoceptive inference in emotional states and MDD^25^. For example, vagus nerve stimulation^26^ brings about treatment response by modifying the “autonomic depression loop,” causing enhanced neuroplasticity and changes in neuronal firing patterns in higher brain regions^27^. Similarly, early studies with botulinum toxin, which blocks the afferent input from the face, appear to show preliminary benefit in MDD^19,28^. However, these treatments are invasive and difficult to access.

FES is a technique in which muscles are electrically stimulated, causing them to contract. FES has therapeutic efficacy for restoration of motor and sensory function post-stroke and spinal cord injury^29,30^, and this process is accompanied by plasticity in the central nervous system^31^. The FES protocol of the zygomatic major and orbicularis oculi for improving symptoms of MDD is rooted in the Facial Feedback Hypothesis^14,32^, suggesting that facial movement could influence emotional experience^33^. Certain facial muscle movements can be easily controlled voluntarily, while others occur primarily during “genuine” emotions. Voluntary smiles (e.g., for social purposes, without any particular emotional involvement) usually consist only of the upward curving of the lips, whereas spontaneous smiles due to positive emotions also involve the eyes (the so-called “Duchenne marker”), characterized by a rising of the cheeks and the appearance of crows-feet wrinkles next to the eyes^34,35^. These two facial expressions are mediated by different neural pathways: (i) voluntary smiles are initiated in the motor cortex and routed through the pyramidal motor system, whereas (ii) involuntary smiles arise mainly from subcortical nuclei and are routed through the extrapyramidal motor system^15^. An unexpected by-product of this research has been the observation that voluntarily producing and holding an expression can induce the corresponding emotion^36,37^. This effect is more pronounced when a person pays specific attention to voluntarily activating muscles that are usually only used involuntarily (e.g., the Duchenne marker)^38,39^. FES could facilitate the activation and holding of muscles that are typically associated with spontaneous smiles and elicit the corresponding emotion which, when induced in a repetitive manner, has neuroplastic effects^18,19^.

Functional Electrical Stimulation Modulates Emotions in Healthy Individuals

Zariffa et al. (2014) conducted a preliminary proof-of-concept study with transcutaneous facial FES of the “Duchenne” muscles to examine the ability of a single session to modulate mood-related effects.^10^ They hypothesized that the FES might enhance the mood-related effects of voluntarily activating facial muscles with close neural connections to the subcortical nuclei regulating emotions, such as the amygdala. Twelve healthy subjects, who received the FES and were asked to voluntarily move the target muscles at the time of stimulation, were compared to 12 subjects in the control group, who performed the same procedure but without any stimulation. Study outcomes were the scores on the Positive and Negative Affect Schedule-X (PANAS-X),^80^ which asks a participant to rate 60 words/expressions describing feelings on a scale of 1 to 5, depending on how strongly they feel a particular emotion while completing the assessment. Those who received the FES reported changes in the “determined,” “daring,” “scared,” and “concentrating” scores of the PANAS-X, which suggested the possibility that emotions relevant to MDD could be modulated by FES.

Functional Electrical Stimulation Improves Symptoms of Major Depressive Disorder

Kapadia et al. (2019) conducted a follow-up open-label mixed-methods study on individuals with MDD, where they wanted to explore whether 10 sessions of the FES would lead to improvements in depressive symptoms.^11^ This study recruited 10 individuals with moderate-to-severe MDD, and all study participants received facial FES of the “Duchenne” muscles three times per week for a minimum of 10 sessions and a maximum of 40 sessions. All participants received 10 sessions of the FES and were invited to continue therapy for up to 40 sessions if they wished (5 out of 10 participants requested to undergo 40 FES sessions). The results were promising: participants experienced early improvements in depressive symptoms as measured by the Hamilton Depression Rating Scale (HAM-D)^81^ and Inventory of Depressive Symptomology (IDS)^82^ upon completing 10 sessions of treatment. On average, the participants showed an improvement on the HAM-D by 8.1 points (SD = 5.26, p = .005) and on the IDS by 14 points (SD = 11.05, p = .008). Eighty percent of participants showed a reduction by at least 30%, 50% achieved clinical response (i.e., change in the HAM-D by at least 50%), and 60% entered remission. The reported adverse events were insignificant, involving commonly reported redness or skin irritation underneath the stimulation site, muscle soreness, and muscle discomfort. All participants enrolled in the trials adhered to the treatment protocol, showing high compliance. FES was well-tolerated, with the potential to be administered with limited physician oversight.

Neurobiological mechanisms and target circuitry

While patterns of neural activity corresponding to facial expressions are distributed across multiple facial motor brain areas, the neurons of the amygdala play a key regulatory role. The amygdala has a mirror neuron system that monitors the facial expression itself, as well as the autonomic responses that accompany emotional expressions.^83-85^ Cellular studies have shown that the onset of the firing rate of amygdala neurons occurs mainly after the onset of the muscular activity corresponding to the movement of a facial muscle, thus supporting the existence of the ascending segment of the emotion-to-motor transformation loop and the involvement of the amygdala in the sensory monitoring of generated facial expressions.^83, 86^ Other pieces of evidence point to the role of interoceptive afferents projecting to the amygdala and the midcingulate through the insula via the glossopharyngeal and vagus nerves.^87, 88^ The trigeminal nerve is also involved - the proprioceptive signals from the muscle fibres travel through its optic branch to the mesencephalic trigeminal nucleus and the locus coeruleus, which have connections with the telencephalic structures involved in emotional regulation – the amygdala and ventromedial prefrontal cortex.^89^ Therefore, the neurophysiological activity of the facial muscles induced by the FES may send patterned proprioceptive and interoceptive bottom-up inputs to the amygdala through the cranial nerves and the brainstem, which will lead to neuroplastic changes in the emotion-to-motor transformation loop.

The existence of such loops has been supported by emerging botulinum toxin studies paired with fMRI,^75, 90^ where the induced paralysis of frown muscles interrupted the activity of such circuitry and dampened emotional distress signals associated with frowning in depression. This led to improvements in mood and the decreased activity of the amygdala,^75, 90^ and the baseline activity of the amygdala was restored after the effect of neuromuscular treatment had worn off.^90^ Extensive work on neuroimaging MDD correlates^91, 92^ suggests that amygdala activity and functional connectivity are robust and replicable biomarkers of depressive symptoms and antidepressant treatment response. Amygdala is also involved in processing social and cultural information about others, which is retrieved from their facial expressions.^93, 94^ Based on this evidence, depressive symptoms and low mood can be improved in a “bottom-up” fashion by modulating facial muscles through the amygdala circuitry, which is congruent with the effects of FES on neuroplasticity.

Dosing, adverse effects, and contraindications

To understand the optimal dosing or adverse events associated with the FES, certain studies have shown that a minimum of 10 FES sessions were needed to detect a change in function, and 40 FES sessions were needed to see a change in the quality of life (17). Regarding the tolerability and safety of the FES, no significant adverse events have been noted. Common mild adverse events include redness below the area of the electrodes, which typically dissipates within 24 hours, and occasional muscle fatigue or soreness, which also typically resolves without intervention within 24 hours.^25, 52^

The use of FES in a therapeutic setting is associated with certain contraindications, which include metal implants at the stimulation site, pacemakers, open wounds or rash at the electrode placement site, and uncontrolled autonomic dysreflexia.^53^ The FES target muscles have to be accessible for placement of the stimulation electrodes – for example, transcutaneous FES can be delivered with a greater level of specificity when the target muscles are more superficial (e.g., flexor carpi ulnaris, flexor carpi radialis), while for deeper muscles, eliciting an isolated contraction without stimulating the overlying muscles (e.g., flexor digitorum profundus) might be more challenging. Certain muscles, such as the iliopsoas*,* cannot be stimulated using surface electrodes due to their anatomical location. Furthermore, the degree of lower motor neuron injury or nerve root damage of the stimulated muscle must not be significant. Finally, the patient must be cognitively able to follow the instructions and actively participate in the therapy process for the maximal therapeutic benefit from the FES.

Following a study on the safety and feasibility of FES among 12 healthy participants (15) and an open-label study with 10 MDD participants (17), with 80% of participants showing a clinically significant reduction in depressive symptoms, we believe it is now necessary to conduct a pilot sham-controlled trial that will provide the required evidence and clinical parameter estimates to plan a larger sham-controlled trial.

# 4. Study Objectives

Based on the previous open-label feasibility study (17), our overarching goal is to enhance positive emotions and treat MDD by 20 sessions (over 20 days, with one sessions per day) of FES of the Duchenne muscles in participants with MDD in a randomized controlled trial (RCT). The other primary objectives of the trial will be to estimate the tolerability, and safety outcomes of recruitment, dropout, data completion, and protocol compliance rates, and the number and nature of adverse events and serious adverse events, to evaluate patient experience with the FES. In this RCT, 60 participants with non-treatment resistant MDD will be randomized to receive twenty 60-minute sessions of FES vs. sham-FES.

**Primary Objectives**

**Aim 1:** to evaluate the efficacy of FES for MDD

**Hypothesis 1:** over 4 weeks (20 sessions), participants randomized to FES will have a significantly larger reduction in the HAM-D-17 total score than those randomized to sham-FES.

**Aim 2:** to evaluate the tolerability, and safety of the FES for MDD

**Hypothesis 2a:** the overall dropout and protocol compliance rates will not differ significantly between participants randomized to FES or sham-FES.

**Hypothesis 2b:** the overall number of adverse events (AEs) and serious adverse events (SAEs) will not differ significantly between participants randomized to FES or sham-FES.

**Hypothesis 2c:** the nature of AEs and SAEs and the patient experience will not have a significant impact on the dropout and protocol compliance rates.

**Secondary Objectives**:

**Aim 3:** to evaluate the response and remission rates; the impact of FES on insomnia, quality of life, and function; and sustainability of effects up to 4 weeks (28 days) after completion of the intervention.

**Hypothesis 3:** Participants randomized to FES will have a significantly larger reduction in the total score of the Quick Inventory of Depressive Symptomatology (QIDS-SR), Generalized Anxiety Disorder-7 Scale (GAD-7), World Health Organization-5 Well-Being Index (WHO-5), and Pittsburgh Sleep Quality Index (PSQI) than those randomized to sham-FES.

**Aim 4:** to explore changes in facial expressivity during semi-structured emotional recall interview, using objective facial coding.

**Hypothesis 4:** Participants randomized to active FES will demonstrate increased frequency, intensity and duration of Duchenne smiles compared to the sham group during emotional recall interviews, reflecting enhanced emotional expressivity.

# 5. Subject Selection and Recruitment

## 5.1. Eligibility Criteria

### Inclusion Criteria

Patients will be included if they:

1. Meet the DSM-5 (67) criteria for unipolar MDD with a current MDE without psychotic features, with ≤ 2 failed treatment trials (non-treatment-resistant depression), validated by MINI (68) done by a trained research assistant.
2. Have no change in the medication regimen or other forms of treatments (e.g., psychotherapy) for at least 4 weeks (28 days) prior to beginning the study and have no plan to change them during the 20-session treatment period (14 days), and the 4-week post-treatment observation period. This will be established through self-report, in combination with the ATHF (69, 70) filled out by the participant.
3. Have an MDD diagnosis as confirmed by the MADRS (71) score of ≥7.
4. Age group between 18 and 70 years of age

### Exclusion Criteria

Patients will be excluded if they:

1. History of epilepsy or seizures.
2. Damage or dysfunction of facial nerves.
3. Metallic orthopedic implants in the mouth (e.g., plates or screws).
4. Current fibromyalgia or currently receiving or have received rTMS within the last 28 days before the screening.
5. History of treatment-resistant depression (TRD) with history of ECT, Magnetic Seizure Therapy, Intravenous Ketamine use in the past or failure of >2 antidepressant treatments of adequate duration and dose during the current episode.
6. Past or current symptoms of mania, hypomania, mixed episodes, psychotic disorders, obsessive-compulsive disorder, post-traumatic stress disorder, active substance abuse or dependence (excluding nicotine and caffeine), neurodegenerative disease, or dementia. This will be confirmed on the MINI (77) administered by a trained research assistant.
7. Current suicidal intent or plan as demonstrated by a score of ≥4 on MADRS item 10.
8. Unable to understand instructions in English.
9. Unable to produce “Duchenne marker” expression with FES, secondary to any type of neurological condition or previous botulinum toxin treatments of facial muscles.

**Justifications for any exclusions based on race, gender, or ethnicity**: non-English speaking individuals are excluded because the ability to accurately and completely communicate study information, answer questions about the study, and obtain consent are necessary.

## 5.2. Concomitant Medications (CONMED)

Participants will be asked to remain on stable dosages of concomitant psychotropic medications. Changes to treatment regimens or the introduction of psychotherapy, however, will not be permitted during the acute intervention phase of the trial (2-week/ 14 days), 4 weeks (28 days) before the start of the trial, and during the 4-week (28 days) follow-up period. If changes to treatment regimens or the introduction of psychotherapy are determined to be required as per the patient’s standard of care, the required changes will be made.

## 5.3. Method of Recruitment

The single study center will be the Interventional Psychiatry Program at St. Michael’s Hospital, Unity Health Toronto (SMH, UHT). The subjects will be recruited from the Interventional Psychiatry Program at St. Michael’s Hospital, Unity Health Toronto. The study will be advertised through posters at St. Michael’s Hospital, the University Health Network and across the Toronto public transit system (TTC). Additionally, a promotional post will be shared on the IPP’s official Instagram page. The target recruitment rate is a total of 60 subjects recruited over 2 years.

Considering our prior experience with this type of study, we do not anticipate any significant compliance issues. We note that the 5 days per week proposed here exceeds the schedule in the preliminary study by 3 sessions per week with the presence of a sham arm; we have included a conservative 15% dropout rate in our sample size calculations^40^. However, a 5-session per week schedule is well tolerated in rTMS for MDD^41^ and in FES for stroke rehabilitation^30^. Notably, since the intervention is a monitored in-person visit, treatment adherence will be known with 100% accuracy.

## 5.4. Retention Strategy, Costs, and Reimbursements

Our retention strategy includes a payment schedule consisting of a one-time payment at the end of the study. The research staff will also give each participant a reminder call for the initial session and each follow-up session. Each research staff member will be easily available for the participants to contact via email or phone. Participants need to provide informed consent. We will be asking each participant to answer questions about the consent form to determine that the study process and the duration of participation are completely understood by all participants. We will aim to have a specific research team member assigned to complete all sessions with the same participant. The study team will work hard at forming a professional relationship with the participant so they feel comfortable and willing to discuss what may be sensitive information. Participants will no longer be eligible to continue the study if they miss more than 2 stimulation sessions. Participants will be reimbursed $30 per day for in-person visits. The participant will be informed that compensation for their participation will be received at the end of their involvement in the study.

# 6. Study Design

## 6.1. Research design and methods

This is a single-site, pilot, double-blind, randomized, sham-controlled, 20 sessions over 20 weekdays, with one session per day, clinical trial. The trial will evaluate the efficacy, tolerability, and safety of 20 FES sessions for MDE with a primary diagnosis of MDD. We will also collect the data on remission rate of 20 FES sessions for the symptoms of MDD and associated anxiety, quality of life, and sleep. The trial design is described in **Figure 3** and scales & schedule in **Table 1**.

Sixty participants with MDD, with ≤ 2 failed treatment trials (non-treatment-resistant depression) will be recruited from the outpatient clinic. This will include males and non-pregnant females aged between 18 and 70 years with a diagnosis of unipolar, non-psychotic MDD. Eligible participants will have a MADRS score of ≥7 and will have no active suicidal ideation. The study visits will be conducted at St. Michael’s Hospital, Unity Health Toronto. We estimate that it will take two years to complete study enrollment.

Patients interested in the study will be given an option to speak to the study coordinator during a pre-screening phone call, where researchers will provide a brief background about MDD and FES, as well as the time commitment required for the study. Any additional initial questions will be answered at this point, and verbal consent will be obtained before the screening visit and written informed consent will be obtained at the first on-site visit. If the participant is interested in continuing, he/she will be asked to go through a screening survey through REDCap which will be a preliminary measure of their eligibility for the study. Before participants begin answering the survey questions, they will be asked to confirm their agreement to complete this survey. If the participant appears eligible, a screening visit will be scheduled to happen over the phone. At the beginning of the screening visit, participants will again confirm their consent to participate in this study. The date of this verbal consent will be documented in the ICF. Written consent will be obtained at first on-site visit and a signed copy of the ICF will be provided to each participant.

Eligible participants enrolled in this clinical trial will have a total of 6 virtual visits including one screening visit (Visit -1), one baseline visit (Visit 0) and four post-stimulation visits (Visits 21-24). They will also have 20 on-site FES treatment visits (Visits 1-20) over 20 days, with one session per day. The screening Visit will take about 90 minutes, and the baseline Visit will take (Visit 0) will take approximately 45 minutes, and treatment days (Visits 1-20) will take 60 mins each (45 min for stimulation and 15 min for preparations). Sessions 5, 10, 15, and 20 will last about 105 minutes and will include similar clinical assessments/questionnaires to the baseline visit. A 5-minute emotional interview will be conducted at baseline (before Visit 1) and after Visits 5, 10, 15, and 20, with facial expressions video-recorded. The post-stimulation visits will take approximately 45 mins. We estimate that the total participation duration will be approximately 38 hours.

Prior to the baseline visit, participants will undergo a screening evaluation that includes a medical/treatment history assessment and a confirmation of the MDD diagnosis. If all inclusion criteria are met, the baseline visit will be scheduled. During Visit 0, medical assessments will be performed, and baseline assessments consisting of a semi-structured interview and self-report scales will be conducted. Once all baseline measures are collected, participants will be randomly assigned to one of two treatment arms:

- **ARM 1. 40 Hz FES:** 40 Hz Functional Electrical Stimulation (FES) stimulation, 45 mins per day for the duration of 4 weeks (20 sessions over 20 days, with one session per day).
- **ARM 2. Sham FES:** 45 mins per day for the duration of 4 weeks (20 sessions over 20 days, with one session per day).

Patients who miss a treatment visit, including due to statutory holidays, will be flagged in the database as non-adherent but will not be withdrawn from the study. They will still receive the entire treatment schedule but over a longer time period. However, if subjects miss more than two treatment visits during treatment visits 1-20, they will be withdrawn.

Depressive symptoms will be assessed using the HAM-D-17 and the self-rated 16-item Quick Inventory of Depressive Symptoms (QIDS-SR-16). All the self-report questionnaires will be done through REDCap and an independently trained rater will administer the psychiatric interview at baseline (Visit 0), at the end of each treatment week (Visits 5, 10, 15, and 20), and during the post-stimulation visits (Visits 21 to 24). The independent raters will be blinded to the treatment being administered. All treatments will be conducted with minimization of personal contact (i.e., verbal communication) with the subject to reduce the impact of nonspecific therapeutic contact on outcomes.

Additionally, patients who participate in the study will not be allowed to meet with other participants before, during, and after study procedures in order to maintain the blind. Patients will be discontinued if they experience worsening in depression, where worsening of depression is defined as an increase in the QIDS-SR-16 score from baseline by more than 25% during two consecutive assessments or development of active suicidal intent or attempted suicide.

**Figure 2:** Study Design

**Table 1:** Study Schedule

## 6.2. Randomization and Blinding

Participants will be randomized in a 1:1 allocation to receive FES (active pattern) or FES (sham pattern). Block randomization will be computer-generated, and the samples will be stratified for sex, given the potential differences in response and tolerability in these groups. A biostatistician will generate the randomization schedule^48^, to be administered by a research assistant who is not associated with the study and instructed not to divulge group assignments to the participants to allow for concealment.

Participants and most study staff will be blinded. Only the physical therapist or a trained research staff will need to monitor the participant. Measures taken to prevent functional unblinding include sensory and non-patterned sham and delivering automated messages to both active and sham groups. We will also evaluate each participant’s ability to discriminate what treatment they received after each session to assess blinding effectiveness^49^.

We will randomize subjects to study arms (20 sessions active or 20 sessions sham) using a secure online randomization module on REDCap. The block size will be fixed, and study personnel will be blinded to the randomization block size. An independent assistant, external to the study, will manage the randomization of subjects. The subject number and treatment code will be assigned after the subject details have been obtained. Based on this information, the independent assistant will assign a unique subject number and treatment code, which will dictate the treatment assignment for the subject. An unblinded code sheet that matches these stimulation codes to the treatment arm will be kept by an external coordinator and will not be available to the study coordinator or research assistants. The unblinded code sheet will have the following information:

1. The initial identifier codes for all potential participants

2. Stimulation code: 6-digit numerical code for the stimulation session

3. Condition number: Numerical code for the condition

4. Condition name: Name of the condition

A copy of this code sheet with the condition name removed will be provided to the study coordinator and research assistants. These linked codes ensure that the study coordinator and research assistants are kept blinded to the type of treatment each participant will receive.

## 6.3. Study Schedule

It is important to note that consent, scales, and experiments will all take place in a private room. Any phone calls will take place in a private lab environment as well.

Screening Visit

***Individuals who are referred by a mental health care provider or will approach the study team through flyers:***

Individuals who are referred by a mental health care provider will be contacted via phone for a pre-screening call to give them additional background information on the study and associated participant responsibilities. Researchers will keep a Telephone Contact log (within the Master Linking Log) for each telephone conversation with a participant throughout the study. There will be an entry for each participant, and the log will be filed in the participant binder. If a participant is determined to be ineligible, data from the screening will be retained. However, they have the right to request the withdrawal of their screening data at any time, as long as the data has not yet been anonymized or included in aggregated analyses.

During the telephone pre-screening, researchers will provide a brief background about MDD and FES. Any initial questions will be answered at this point. The participant will be asked if they have any additional questions. Once all questions have been answered, the participant will be asked if he/she is still interested in participating in the study. If yes, the researcher will obtain verbal consent and ask them to confirm their email address. They will access a screening survey through REDCap via a link which will be sent to their email.

***Individuals who will be directed to the REDCap survey via a link provided in the social media advertisements:***

Upon clicking the link, they will first be taken to a landing page that outlines key study information, including the study’s purpose and timeline, potential risks and benefits, participation and withdrawal details, and confidentiality measures. Contact information will be available both in the advertisement and at the bottom of the landing page for the ones who have questions about the details.

For all the individuals who will complete the REDCap survey, the survey will be a preliminary measure of their eligibility for the study. Once a participant has completed the REDCap screening form, participants who appear eligible will receive another telephone call to schedule the screening visit for them. The written informed consent will be obtained at the first on-site visit.

The following procedures will be completed at the screening visit over the phone:

### Inclusion/exclusion review

### Demographic review

### Review of medical, psychiatric, and medication history, including review and confirmation of diagnosis

### FES safety questionnaire

Additionally, female participants will be asked to confirm they are not pregnant, and they are not planning to become pregnant throughout the duration of the study. If the subject satisfies the inclusion criteria, the baseline Visit (Visit 0) will be scheduled, and a reminder call or e-mail will be given at least 24 hours before that Visit.

Baseline Visit (Visit 0)

A baseline assessment will take place before the first FES visit. The research team will assess depression, sleep, and quality of life over the phone at baseline.

The remainder of the activities will be planned according to schedule, resources, timing, and subject tolerability. A baseline assessment may happen on the first day of stimulation prior to the FES (e.g., a questionnaire). The baseline visit includes:

- Psychiatric interview
- HAM-D-17
- Self-report scales (this information will be recorded in REDCap)
- QIDS-SR-16
- GAD-7
- WHO-5
- PSQI
- Assessment of CONMED record
- Assessment of FES Expectancy

FES Visits (20 Visits)

The FES study visits will be conducted at St. Michael's Hospital. Participants will be randomized to undergo either 20 days (weekdays) of FES once a day (20 visits) or 20 days of sham FES once a day (20 visits). Participants will be allowed to miss up to 2 treatment visits. Additional visits will be added on to reach the expected number of visits if it is within a reasonable timeframe as determined by the investigator.

The FES treatment sessions will be 60 minutes in duration (45 min for stimulation and 15 min for preparations), delivered 5 times/week, for a total of 20 FES sessions. Each day will consist of the following:

- A review of FES side effects and adverse events will be completed daily before and after stimulation.
- 45 mins of FES stimulation to target Duchenne muscles, while doing the task described under section #7 (Trial Intervention).
- Participant tolerance
  - Subjects will be queried each day about their experience and how they are doing. If participants or their family members express that the participant is having difficulty, the study staff will work with them to reduce any possible burden.
  - A participant “FES Experience” Assessment will be completed with the subject at the end of each week (Visits 5, 10, 15, and 20) to monitor the participant burden.
- Psychiatric interview - will be completed with the subject at the end of each intervention week (sessions 5, 10, 15, and 20).
- HAM-D-17
- Self-report scales - will be completed by the subject daily after treatment and will be received via email:
- QIDS-SR-16 using REDCap
- Self-report scales - will be completed with the subject at sessions 5, 10,15, and 20 using REDCap:
- GAD-7
- WHO-5
- PSQI
- Assessment of CONMED record - will be completed with the subject at sessions 5, 10, 15, and 20.
- Assessment of FES Expectancy - will be completed with the subject at sessions 5, 10, 15, and 20 using REDCap.

Semi-structured emotional recall interview: A 5-minute emotional interview will be conducted at baseline (before Visit 1) and after Visits 5, 10, 15, and 20, with facial expressions video-recorded. The video recordings are mandatory, and participants' smiles should be detectable, but only to be viewed by the study personnel.

Post-Stimulation Follow-Up Visits (Visits 21-24)

Visit 21 will take place within 1 week after Day 20 of treatment. The study team will aim to schedule the appointments as close as possible to the last FES study visit (Session 20- Day 20). Subsequent visits will take place two, three, and four weeks after Day 20 of the intervention. Each follow-up visit will consist of the following:

- Psychiatric interview
- HAM-D-17
- Self-report scales using REDCap
- QIDS-SR-16
- GAD-7
- WHO-5
- PSQI
- Assessment of CONMED record
- Assessment of FES Expectancy- will be completed with the subject using REDCap
- Review of FES side effects and adverse events

If participants do not complete the entire study, we may ask them to return to St. Michael’s Hospital for some of the follow-up visits, including a psychiatric interview, self-report scales, and/or neuropsychological testing.

## 6.4. Clinical Scales and Self-Report Measures

All clinical assessments will be completed by a rater blinded to the treatment arm, or participants themselves (self-report scales). On visits with both the clinical scales and treatment, the clinical scales will be completed after treatment. The rater will not be otherwise involved in the study. If the rater’s blind is broken, it will be documented, and efforts will be made to find a substitute rater.

- Montgomery Åsberg Depression Rating Scale (MADRS)^42^

The clinician-administered MADRS will be used as part of the screening process. This is a 10-item clinician-rated scale designed to measure depression severity and detects changes due to treatment. Participants will need to obtain a minimal total score of 7 total at screening to be eligible for inclusion.

- Hamilton Depression Rating Scale 17-Item (HAM-D-17)^43^

The HAM-D-17 is a semi-structured, clinician-administered interview that has been well-validated in measuring the presence and severity of depression. This scale will be administered at baseline, at the end of each treatment week, and at each follow-up visit.

- 16-Item Quick Inventory of Depressive Symptomatology-Self Report (QIDS-SR-16)^44^

The Quick Inventory of Depressive Symptomatology-Self Report (QIDS-SR-16) is a 16-item, self-rated survey that has been psychometrically validated to screen for depression, using the diagnostic criteria for MDD from the DSM-IV. This self-report measure will be administered at baseline, during each treatment visit, and at each follow-up visit. The QIDS-SR-16 will be completed with the subject using REDCap.

- General Anxiety Disorder (GAD-7)^45^

The GAD-7 is a 7-item self-rated questionnaire for assessing generalized anxiety disorder and its severity. Items are ranked on a 4-point scale from 0 (not at all sure) to 3 (nearly every day), providing a total severity score from 0 to 21. This self-report measure will be done at baseline, during treatment visits 5, 10, 15, and 20, and at each follow-up visit using REDCap.

- World Health Organization-5 Well-Being Index (WHO-5)^46^

The WHO-5 Well-Being Index is a 5-item self-rated scale that is designed to measure well-being over the past two weeks. Participants will rate the frequency or consistency of each positive feeling on a 6-point scale from 0 (at no time) to 5 (all of the time). The sum of scores from the five items will then be multiplied by 4, representing the participant’s perceived quality of life as a percentage. This self-report measure will be done at baseline, during treatment visits 5, 10, 15, and 20, and at each follow-up visit using REDCap.

- Pittsburgh Sleep Quality Index (PSQI)^47^

The PSQI is a self-rated scale containing 19 items that measure sleep habits and quality.

This scale is broken up into seven components that assess sleep quality, latency, duration, efficiency, disturbances, medication use, and daytime dysfunction. The sum of scores from all seven components will generate a global score of 0-21, with higher scores indicating lower sleep quality. This self-report measure will be done at baseline, during treatment visits 5, 10, 15, and 20, and at each follow-up visit using REDCap.

- Emotional Recall Interview (video-recorded)

Participants will complete a brief, semi-structured emotional recall interview (5 minutes) at baseline and the end of each treatment week. The interview involves describing personal positive memories in response to open-ended prompts. Sessions are video recorded for later analysis using automated facial coding software to quantify Duchenne markers. This method will be used to assess changes in facial expressivity.

## 6.5. REDCap

Research Electronic Data Capture (REDCap; https://www.project-redcap.org) software supported by Applied Health Research Centre (AHRC) at UHT, will be used for data collection and overall study data management over the course of this project. REDCap is an open-source, web-based clinical data management and electronic data capture system and database. The system is developed and managed in compliance with UHT privacy, the Health Insurance Portability and Accountability Act, the Personal Information Protection and Electronic Documents Act, and Food and Drug Administration 21 Code of Federal Regulations Part 11 regulations, providing functions such as defined user roles and privileges, user authentication and encryption for in-transit data, de-identification of protected health information and comprehensive auditing features to record and monitor access and changes to data. This system will be used to send scheduled questionnaires to participants and store active data and data monitoring and for the query and export of datasets for statistical analysis and modeling.

Access to REDCap will be secured through a secure web portal and protected by multiple levels of authentication. A Project Coordinator will be assigned the project administrative privileges for study configuration, data collection management, and quality control.

Upon acceptance to the study, participants will complete mental health questionnaires on a daily and weekly basis. To complete these questionnaires, participants will receive an email notification with the REDCap survey link to their personal email address. Upon receiving the link, participants will answer the self-report questionnaires provided through the link. Active data will be sent to and stored on the REDCap server and will be linked by the unique ID for final analysis. Access to this data will only be granted to the project administrator to download the data from the REDCap server for analysis.

# 7. Trial Intervention

## 7.1. FES Active (motor and sensory stimulation):

Participants allocated to the active arm will receive simultaneous transcutaneous FES therapy of bilateral zygomaticus major and orbicularis oculi muscles, which produce an expression of happiness according to the Facial Action Coding System (60). Surface self-adhesive electrodes will be placed to the muscles as in Figure 3. FES will be delivered simultaneously to all four muscles using the Twin Stim® Plus Digital TENS/EMS FES device. FES stimulation parameters to be used are 300μs long charge-balanced biphasic pulses delivered at 40 Hz, with amplitudes in the range of 8-15 mA, and alternating periods of stimulation and rest of 15-sec duration each; these are stimulation values commonly used in FES studies for muscle activation (29).

Synchronized stimulation of four muscles (i.e., bilateral zygomaticus major and bilateral orbicularis oculi) will be delivered in a cycle consisting of 15 sec ON and 15 sec OFF, with “smile and hold” instructions provided only during 15 sec ON time. While receiving FES:

- Participants will watch videos with positive/comedy content. The videos consist of comedy films without violent content. During the preparation period, participants may select a film from a collection we provide for them. During the visit, they can ask us to change the video If they do not find the selected one funny.
- Also, as done in the pilot study^17^, participants will be reminded to attempt to voluntarily produce the desired expression (smile with the Duchenne marker^34^) during FES. Participants receive on-screen prompts to “smile and hold”. Research staff reiterate the instruction if needed. After each executed smile, participants press a response button; upon pressing, an indicator changes from red to green to confirm receipt. Participants must press the button at least once per minute for the duration of the task. Failure to press the button for three consecutive minutes triggers an automatic pause and a brief comprehension check to ensure the instruction is understood before resuming.

Instructions for Duchenne smile accompanied with the videos:

a. Relax your face completely
b. Gently lift the corners of your mouth upward
c. At the same time, narrow your eyes slightly, letting the skin at the outer corners of your eyes wrinkle a bit, and let your cheeks rise.
d. Keep both the mouth corners raised and the cheeks/eyes engaged together — this should feel like a warm, genuine smile.
e. Hold this expression for about 15 seconds while breathing normally.
f. Now press the button to resume the video

- All FES visits will be video recorded to verify that the intended smile pattern is elicited, to confirm that each button press corresponds to an actual smile response, and to facilitate more accurate analysis. We will use a study-dedicated Canon EOS digital camera (not a personal phone) to record the videos. The camera saves recordings to its memory card. Immediately after each visit, the videos will be transferred to a password-protected external hard drive, and the files will then be deleted from the camera’s memory card.

**Figure 3:** Cutaneous electrode placement for the functional electrical stimulation of bilateral zygomaticus major and orbicularis oculi muscles for the treatment of Major Depressive Disorder (left). The target “Duchenne” muscles are emphasized in orange, and the facial nerve is emphasized in magenta (right). Created with Complete Anatomy, 3D4 Medical.

## 7.2. FES Sham (Sensory & non-patterned stimulation):

Participants recruited to the sham arm will also receive FES of the facial muscles in the same setup as the experimental group, but with only sensory stimulation (in the range of 1-8 mA stimulation – just enough to feel the stimulation, with other parameters the same as active arm) and in a non-Duchenne smile pattern. Sham group will also watch the videos with positive content and will receive the instructions for the Duchenne smile.

Though we cannot be certain that this sham stimulation will be completely ineffective, the premise of the current trial is that facilitating the activation of “Duchenne muscles” via patterned stimulation will result in the reinforcement of neurological pathways responsible for mood elevation. This control group of non-Duchenne pattern stimulation will allow us to determine if the Duchenne Marker is key to improving patient outcomes. Further, this sham design is informed by guidelines for sham stimulation for neurostimulation studies41,51,52.

## 7.3. Study Investigational Product

### 7.3.1. Device Overview

The Twin Stim® Plus Digital TENS/EMS (HC License name: TWIN STIM SERIES) can be used both with the battery and plugged in. In this study, the plug in will be used because the device does not need to be portable, the battery charge will not be a variable/concern, and to access the battery compartment would need to disassemble a component of the stimulation system. The unit sends electrical impulses via electrodes to the body and reach the nerves and underlying muscle group. This unit is a combination stimulator of TENS and EMS which can be used for muscle stimulation and pain relief. The device is provided with four controllable output channels, each independent of each other. An electrode pair can be connected to each output channel. The intensity controls are protected by a cap to avoid accidental touch. The settings are controlled by pressing buttons.

### 7.3.2. Operation of the device

The Twin Stim® Plus Digital TENS/EMS system includes a Stimulator, Stimulator Power Supply and Cord, Hand Switch, Electrode. Technician can turn on the stimulator by pressing ON button firmly for 1 second and select the desired protocol and press Begin Session. They can also finish the protocol by turning off the device.

### 7.3.3. Device Safety Features

For safety reasons, the Twin Stim® Plus Digital TENS/EMS device should be checked for any external damage, defective operating elements, and the usability of accessories (including cables).

# 8. Outcome Measures

## 8.1. Primary Outcome Measures

The primary outcome will include the A) change in depression symptoms, measured by the HAM-D-17 scale^53,54^, and B) the tolerability, and safety outcomes that include the recruitment, dropout, data completion, and protocol compliance rates, as well as the number and nature of AEs and SAEs. Self-report questionnaires will be completed via REDCap.

## 8.2. Secondary Outcome Measures

Secondary outcomes will be A) changes in anxiety, measured by the Generalized Anxiety Disorder-7 (GAD-7)^56^; B) changes in quality of life, measured by the World Health Organization Quality of Life Questionnaire (WHO-5)^57^; C) change in subjective sleep, measured by the Pittsburgh Sleep Quality Index (PSQI)^47^; D) HAM-D-17 response and remission rates and sustainability of effects for up to 4 weeks (28 days) following the intervention. The choice of scales in this study has been informed by our pilot study^17^; we have added the FES experience scale.

The MADRS will be used for recruitment, and the HAM-D-17 will be used for the evaluation of treatment response as per current best practice in clinical trials. Self-report questionnaires will be completed via REDCap.

In addition, an exploratory outcome will include analysis of facial expressivity during emotional recall interviews. Recordings will be analyzed using automated facial coding software (OpenFace) to quantify Duchenne smile metrics, including frequency, duration and intensity.

# 9. Potential Risks and Benefits

## 9.1. Potential Benefits

MDD is a common, severe psychiatric illness. However, participants in this study may experience some degree of relief from mood symptoms as a result of the FES intervention. There are no serious risks to the participant from the treatment used in this study. The chance to understand and develop a new treatment for a wide range of psychiatric disorders is an important step in helping the millions of people in the world who suffer from mental illness.

## 9.2. Potential Risks and Analysis of Risk/Benefit Ratio

Functional Electrical Stimulation (FES)

In our initial studies of FES in both healthy and MDD participants^15,17^, we saw (i) no safety concerns and (ii) no AEs. Historically, transcutaneous FES has been extensively used in neurorehabilitation and AEs reported are rare and mild, such as redness/irritation of the skin underneath the stimulation site, muscle soreness, and muscle discomfort^15,17,29^. We now propose to apply FES 10 times a week – 3 times more/week than in the pilot study for MDD, but we do not foresee any major risks/safety concerns as alluded to above. We will maintain an AE self-report and Adverse Event Log (AEL) as part of the study, to be systematically completed by the study coordinator.

Clinical Assessments

Self-report assessments contain questions regarding sensitive personal information. This risk is necessary in order to assess mood symptoms and associated psychopathology. Subjects will be assured upon intake that only study personnel will see any rating form responses.

Video-recorded Emotional Interview and OpenFace Program:

The emotional recall interview involves discussing personal positive memories in a semi-structured format, which may occasionally evoke unexpected emotional reactions or psychological discomfort. Although the task is designed to be uplifting and non-invasive, participants may find recalling emotional experiences to be mildly distressing. Participants will be reminded that they can pause or discontinue the task at any time, and support from study personnel will be available if any discomfort arises.

Additionally, during the emotional interview, participants may accidentally share identifying information about themselves or others (for example, full names, workplaces, or specific personal details). This creates a risk of inadvertent disclosure of identifiable information in the recording. Because the interview is recorded, the file itself contains identifiable features. To reduce these risks:

-	Participants will be instructed not to mention full names, addresses, workplaces, or other specific identifiers during the interview.

-	Interviewers will redirect the participant if such details are shared.

-	Recordings will be stored only on secure password protected hard drive with restricted access.

-	Only de-identified, numeric facial-movement data (linked to study IDs, not names) will be used for analysis or publication.

Notably, although OpenFace uses machine-learning algorithms to analyze facial movements, it does not perform facial recognition. The privacy risk comes from the identifiable video, not from the OpenFace output. OpenFace analysis is performed locally, and videos are not uploaded to any external service.

Reproductive Risks

For female participants who are able to conceive, FES stimulation during pregnancy has no known risks on the ova or fetuses. However, there is always a possibility that if participants are pregnant, FES stimulation may have risks that we do not know about. For this reason, such participants should not participate in the study if they may be pregnant.

Appropriate family planning methods will be discussed with participants and decided upon in consultation with the study doctor. To confirm that a female of childbearing potential is not pregnant before starting the study, the RC will ask females if they are pregnant or planning to become pregnant throughout the duration of the study. If they become pregnant during their involvement in the study, they should immediately notify the investigators.

# 10. Data and Safety Monitoring

## 10.1. Quality Assurance

Methods for quality assurance will be strictly observed. A start-up meeting involving all study site RCs, co-investigators, and partners will be held prior to the study launch. Further training will be held at the site prior to commencing the study. During training, great care will be taken to fully explain the study procedures and case report form (CRF) completion.

## 10.3. Safety Oversight

Methods for quality assurance will be strictly observed. A start-up meeting involving all study site RCs, co-investigators, and partners will be held prior to the study launch. Further training will be held at the site prior to commencing the study. During training, great care will be taken to fully explain the study procedures and CRF completion, as well as sample handling procedures.

## 10.4. Early Withdrawal

Participants are free to withdraw their consent and stop research study participation at any time without penalty or loss of benefits to which they are otherwise entitled. A participant’s doctor may withdraw them from the study if he/she feels that it is in their best interest. The Principal Investigator may also decide to terminate the study at any time if the stimulation device is believed to be unsafe.

A study participant will be discontinued from further participation if:

- The participant fails to adhere to the study procedure.
- The participant decides not to be in this study.
- The participant misses more than two treatment visits.
- They experience worsening in depression, defined as an increase in QIDS-SR-16 score from baseline of more than 25% during two consecutive assessments, or development of active suicidal intent or attempted suicide.
- The participant meets any exclusion criteria (either newly developed or not previously recognized).
- Anything, in the opinion of the investigator, would place the participant at increased risk or preclude the participant’s full compliance with or completion of the study.

If a participant withdraws or is withdrawn from the study, they will be asked questions about their experience with the FES stimulation. They will also be asked to cooperate with whatever laboratory tests or medical examinations the doctor considers necessary.

We will collect safety data on any participant discontinued because of an AE or SAE. In any case, every effort will be made to undertake protocol-specific follow-up procedures. If an AE has been reported, researchers will help the participant seek the medical care they need, and a follow-up will be performed by the Principal Investigator. In the case of an early withdrawal, the researcher will make a note to file indicating this.

## 10.5. Study Termination

The study will be stopped (at least temporarily) if studies provide evidence that the FES causes any serious effects on subjects, either short-term or long-term. Examples of findings that might trigger a safety review are the number of SAEs overall, the number of occurrences of a particular type of SAE, severe AEs/reactions, or increased frequency of events.

This study may be prematurely terminated if, in the opinion of the investigator, there is sufficient reasonable cause. Circumstances that may warrant termination include, but are not limited to:

- Determination of unexpected, significant, or unacceptable risk to participants.
- Insufficient adherence to protocol requirements.
- Data that are not sufficiently complete and/or evaluable.
- Plans to modify, suspend or discontinue the development of the study device.

The Research Ethics Board (REB) will be informed promptly and provided the reason(s) for the termination or suspension by the Principal Investigator or institution, as specified by the applicable regulatory requirement(s).

# 11. Safety and Reporting

## 11.1. Safety Parameters

### 11.1.1. Adverse Event Monitoring

AE data will be collected from the start of the experimental protocol to the end of study participation. All AEs, regardless of attribution to the FES or pre/post assessments, will be collected and recorded using a standard AE form. Participants will be asked, in an open-ended way, about the presence of any such AEs on a daily basis. Additionally, a standard questionnaire for FES-related AEs will be completed after every FES session. Intensity of each AE will be graded as mild, moderate, or severe. If an event occurs that is not expected (e.g. is not described in the research protocol or consent form) that indicates a change from baseline in cognition and/or requires immediate attention, such as a seizure, the study MD (or covering investigator) will be informed in real time to assess the event, advise on immediate care of the participant, and to determine the necessary reporting steps. For any events that are serious or unexpected in nature, severity or frequency as compared to the risks described in the study plan will be reviewed by the principal investigator or designee (e.g. a co-investigator) to determine the relationship of the event to the study. Reportable events will be submitted to the Unity Health REB per determined policies.

### 11.1.2. Data Safety Monitoring Board (DSMB)

### Given that this has a small sample size, no Data Safety Monitoring Committee will be formed.

### 11.1.3. General Safety Plan

A licensed physician, credentialed at Unity Health Toronto, will be available and on-call during all visits. Furthermore, the investigator who holds the FES sessions, will be trained to continually assess participants during sessions to monitor for discomfort, to identify early symptoms of syncope (e.g. sweating, pallor). In addition, participants will be trained to apply basic measures to keep the treatment procedure safe.

## 11.2. Adverse Events (AEs)

### 11.2.1. Adverse Event definition

**Adverse event (AE):** An AE is any untoward medical occurrence in a study participant receiving an investigational medicinal product (IMP) and which does not necessarily have a causal relationship with this treatment. An AE can therefore be any unfavorable and unintended sign (including an abnormal laboratory finding), symptom, or disease temporally associated with the use of an IMP, whether or not related to the IMP.

### 11.2.2. Collection of AEs

The condition of the participant will be monitored throughout the study. At each visit, whether scheduled or unscheduled, AEs will be elicited using the scale of the adverse events. In addition, the Investigator will check the participant records for any documented event.

All AEs, including local and systemic reactions not meeting the criteria for “serious adverse events”, will be captured on the appropriate CRF. If the participant reports several signs or symptoms representing a single syndrome or diagnosis, the diagnosis should be recorded in the CRF. In addition, the AE Report Form will be completed by the study coordinator. The AE Report Form includes the following:

- What is known about the therapy
- What is known about previously reported side effects
- If the AE occurred in temporal relation to the therapy
- Whether or not the AE improves or disappears when treatment is stopped
- Whether the AE is worsening of baseline symptoms
- Whether the AE is related to a concurrent medical condition or medication use

Once complete, this form will be given to the Principal Investigator and Co-Investigators, who will review, comment on, and sign this form. The Investigator will grade the severity of all AEs (mild, moderate, or severe), the seriousness (non-serious or serious), and the likelihood that they were related to the FES stimulation (causality). Completed forms will be placed in the participant’s folder.

In addition, the study coordinator will document any AE occurrence on the AE log, which includes information such as the date of the AE, severity, relationship to the treatment (assessed by the Principal Investigator and Co-Investigators), actions taken, and outcome(s). The log will be reviewed and initialed by the Principal Investigator 72 hours after being completed. All AEs occurring during the clinical trial will be documented appropriately regardless of the relationship to FES. All AEs will be followed to adequate resolution and will be graded for severity and relationship to study treatment. Any medical condition noted at the initial session will be considered at baseline and not reported as an AE unless an exacerbation in intensity or frequency (worsening) occurs.

The Investigator will provide detailed information about any abnormalities and about the nature of and reasons for any action taken as well as any other observations or comments that may be useful for the interpretation and understanding of an AE.

### 11.2.3. Severity of AEs

All AEs will be graded for severity using the following guidelines:

**Asymptomatic:** The participant is exhibiting no symptoms due to this event; no treatment needed.

**Mild:** Event results in mild or transient discomfort, not requiring intervention or treatment; does not limit or interfere with daily activities (e.g., insomnia, mild headache)

**Moderate:** Event is sufficiently discomforting so as to limit or interfere with daily activities; may require interventional treatment (e.g., fever requiring antipyretic medication). In the case of a moderate AE, the medical advisor may recommend an over-the-counter medication.

**Severe and undesirable:** Event results in significant symptoms that prevent normal daily activities; may require hospitalization or invasive intervention (e.g., anemia resulting in blood transfusion).

The grading of an AE is up to the medical judgement of the Investigator and will be decided on a case-by-case basis. Changes in the severity of an AE will be documented with the Note to File document and will be filed in the participant’s folder.

### 11.2.4. Causality of AEs

The Principal Investigator and Co-Investigators will together determine whether an AE is associated with the study treatment. The event will be labeled as not associated if the event is temporally independent of the study treatment and can be explained by external factors, such as major life events. If the event is not explainable by the participant’s underlying condition or external factors, then the Investigator will make a determination of the relationship of the AE with the IMP as follows:

**Probable:** reports including good reasons and sufficient documentation to assume a causal relationship, in the sense of plausible, conceivable, likely, but not necessarily highly probable. A reaction that follows a reasonable temporal sequence from the administration of the IMP; or that follows a known or expected response pattern to the suspected medicine; or that is confirmed by stopping or reducing the dosage of the medicine and that could not reasonably be explained by known characteristics of the participant’s clinical state.

**Possible:** reports containing sufficient information to accept the possibility of a causal relationship, in the sense of not impossible and not unlikely, although the connection is uncertain or doubtful, for example, because of missing data or insufficient evidence. A reaction that follows a reasonable temporal sequence from the administration of the IMP; that follows a known or expected response pattern to the suspected medicine; but that could readily have been produced by a number of other factors.

**Unlikely:** reports not following a reasonable temporal sequence from IMP administration. An event that may have been produced by the participant’s clinical state or by environmental factors or other therapies administered.

**Not related (unrelated):** events for which sufficient information exists to conclude that the etiology is unrelated to the IMP.

**Unclassified:** reports which for one reason or another are not yet assessable, e.g., because of outstanding information (can only be a temporary assessment).

### 11.2.5. Outcome of AEs

The outcome of all reported AEs has to be documented as follows:

1. Recovered, resolved
2. Recovering, resolving
3. Not recovered, not resolved (by Study Completion visit)
4. Recovered, resolved with sequelae
5. Fatal
6. Unknown

## 11.3. Serious Adverse Events (SAEs)

An SAE consists of adverse events that result in death, require either inpatient hospitalization or the prolongation of hospitalization, are life-threatening, result in a persistent or significant disability/incapacity or result in congenital anomaly/birth defect. Other important medical events, based upon appropriate medical judgment, may also be considered Serious Adverse Events if a trial participant’s health is at risk and intervention is required to prevent an outcome mentioned.

**NOTE:** The term ‘life-threatening’ refers to an event in which the participant was, in the view of the reporting Investigator, at immediate risk of death at the time of the event; it does not refer to an event that may hypothetically have caused death had it been more severe.

In deciding whether an AE is serious, medical judgment will be exercised. Thus, important AEs that are not immediately life-threatening or do not result in death or hospitalization but may jeopardize the participant or may require intervention to prevent one of the other outcomes listed in the definitions above should also be considered serious.

All SAEs will be recorded on the Serious Adverse Events Form, documented in the SAE log, and reported to the REB. The SAE Form will be completed by the study coordinator and includes information relating to the onset and nature of the SAE, relationship to the study treatment, seriousness of the SAE, treatment required as a response to the SAE, and outcome. This form will be filed in the participant’s folder at the resolution of the event. The study coordinator will complete the SAE log which includes information such as the date of the event, time at which the study team was informed of the event, details, when the REB was notified, and the date that the SAE form was completed.

## 11.4. Unexpected AEs and SAEs

Unexpected Adverse Events (referred to as Unexpected Problems by the Unity Health REB) will be recorded on the Unexpected AE/SAE log and will include information such as the date of the event, when the study team was informed of this event, details of the event when the REB was notified, and whether the SAE form was completed. The REB will be notified of each unexpected AE that may occur during the study.

Unanticipated AEs involving risks to subjects include any incident, experience, or outcome (including an SAE) that meets all of the following criteria:

- Unexpected (in terms of nature, severity, or frequency) given the research procedures that are described in the protocol-related documents (e.g., the REB-approved research protocol and informed consent document, Investigator’s Brochure, Product Monograph); and/or the characteristics of the research participant population being studied; and
- Related or possibly related to participation in the research (possibly related means there is a reasonable possibility that the event, experience, or outcome may have been caused by the FES; and
- Suggests that the research places research participants or others at a greater risk of harm (including physical, psychological, economic, or social harm) than was previously known or recognized.

If an unexpected AE occurs, the REB will be notified, and the study will be adjusted as needed to protect the health and safety of the participants. Depending on the nature of the unexpected AE, the research protocol, inclusion/exclusion criteria, and informed consent will be changed to reflect the possibility of this event reoccurring. During this time, no new participants will be recruited and the research procedures for currently enrolled participants will be stopped. Each unexpected AE will be recorded and reported throughout the study.

## 11.5. Reporting Procedures

AE/SAE reporting will proceed in accordance with the guidelines of St. Michael’s Hospital and the Unity Health REB (**Figure 4**).

**Figure 4**: Reporting of adverse events as per the guidelines outlined by the REB of the clinical site (St. Michael’s Hospital, Unity Health Toronto, Toronto, ON, Canada).

Reporting to the REB: The study investigator will report to the REB all SAEs and unexpected AEs with a reasonable possibility that the SAE/unexpected AE is related to the research, within **7 calendar days** of the study team becoming aware of the event.

All fatal or life-threatening SAEs that are unexpected AND there is a reasonable possibility that the SAE is related to the research intervention will be reported within **3 calendar days**.

Follow-up reports of the SAE will be submitted to the REB whenever new relevant information regarding the SAE becomes available until the resolution of the SAE.

## 11.6. AEs and SAEs Follow-up

The study coordinator will follow up with the subject within one week of an AE or an SAE.

## 11.7. Auditing

Representatives of St. Michael's Hospital, including the Unity Health Research Ethics Board, may look at the study records and at personal health information to verify that the information collected for the study is correct and to make sure the study is following proper laws and guidelines.

# 12. Data Collection and Data Management

## 12.1. Clinical Data Capture and Management

## Research Electronic Data Capture (REDCap; https://www.project-redcap.org) software will be used for data collection and overall study data management over the course of this project. REDCap is an open-source, web-based clinical data management and electronic data capture system and database. The system is developed and managed in compliance with HIPAA, PIPEDA, and FDA 21 CFR Part 11 regulations, providing functions such as defined user roles and privileges, user authentication and encryption, electronic signatures, de-identification of protected health information, comprehensive auditing features to record and monitor access and changes to data, and a validated software development lifecycle. This system will be used to design electronic case report forms (eCRFs), data entry, data monitoring, and cleaning, and for the query and export of datasets for statistical analysis and predictive modeling.

## Access to REDCap will be secured by an encrypted virtual private network and protected by multiple levels of authentication. A Data Manager will be assigned administrative privileges for study configuration, design of CRFs, data entry management, and quality control. The data management team will include qualified database administrators to program scripted and ad-hoc data queries, data backup, and technical support.

## 12.2. Recorded Videos

The facial expressions will be video recorded during the emotional interview at baseline (before Visit 1) and after Visits 5, 10, 15, and 20. Additionally, all the FES visits will be video recorded. Video recording is mandatory, and participants’ faces (including smiles) will be visible. Videos will be saved on encrypted, password-protected computers and transferred to encrypted, password-protected hard drives at SMH (St. Michael’s Hospital). OpenFace runs locally on a UHT computer. The videos are not uploaded to any external website, company, or cloud service. No data leaves the secure institutional environment.

All records pertaining to the trial will be retained and stored for a period of 7 years as per Unity Health requirements.

## 12.3. Privacy, Security, and Data Confidentiality

The confidentiality of the data collected and the identity of the individuals participating in this study will be strictly maintained. All files pertaining to subjects in the study will be coded numerically. Subject names will not be supplied to anyone not directly involved in the study conduct.

For this research study, required personal health information include name, date of birth, new or existing medical records that include psychiatric or medical conditions, current and past medications, presence of surgical implants, and illnesses or psychiatric procedures that may influence the ability to participate in the study. Some personal health information such as consultation reports, and medical history may be kept as source documents. This information will be obtained from the subject, his/her physician, or his/her medical health record. Source documents will always be kept in a locked filing cabinet to limit access, and in the case of electronic source documents, files will be password-protected and saved in a secure server. However, our CRFs will not contain any personal health information. Only the subject number will be recorded in the CRF, and if the subject name appears on any other document (e.g. laboratory report), it must be obliterated on the copy of the document retained in the Trial Master File or made available for audit.

The personally identifying information, including the recorded videos, will be kept private and confidential in accordance with all applicable privacy legislation, including the Personal Health Information Protection Act (PHIPA) of Ontario. No personally identifying information will be allowed off site in any form, unless required by law.

Study findings will be stored on a computer will be stored in accordance with local data protection laws.  The research coordinator, research assistants, and Principal Investigator will have access to all source documents collected over the course of the study. Subjects will be informed that representatives of other parties (including pharmaceutical companies), Research Ethics Board, or regulatory authorities may inspect their records to verify the information collected and that all personal information made available for inspection will be handled in the strictest confidence and in accordance with local data protection laws. The investigator will maintain a personal subject identification list (subject numbers with the corresponding subject names) to enable records to be identified and retrieved.

OpenFace is an open-source research software developed by academic institutions. It is not owned or operated by UHT. For this study, it is used only to convert each video into numerical measures of facial movement (e.g., amount of smiling over time). The participant’s video is imported into OpenFace to extract Action Unit values and facial landmark data. The output is a CSV file containing numbers linked only to the participant’s study ID. These numeric files are what we will use for analysis. The facial videos will not be uploaded anywhere else. The full video files are stored only on a password protected hard drive at UHT site. OpenFace is installed locally on a UHT computer, and the software processes the videos directly from this secure drive; we do not upload the videos to any external website, cloud platform, or third-party server. Thus, only de-identified numerical outputs (e.g., Action Unit values) are generated and used for analysis. No identifiable video files ever leave the UHT environment.

## 12.4. Source Documents

Source data is all information, original records of clinical findings, observations, or other activities in a clinical trial necessary for the reconstruction and evaluation of the trial. Source data are contained in source documents.

Source data include:

### Unity Health REB Documents

• All REB correspondences are documented

• The study staff is REB approved prior to performing any study procedures

• Adverse events and deviations are reported to the REB per current guidelines and stored appropriately

• All versions of the REB protocols and informed consent forms are on file

### Informed Consent

• Ensure that participant identification is not recorded on the informed consent form (ICF) (i.e., no participant ID)

• There is documentation that the participant is given a copy of the consent form

• The participant and study representative signed and dated the consent form for him/herself

• The participant initialed and dated all appropriate pages on the informed consent form

## 12.5. Data Management Responsibilities

The responsibilities designated to each member of the research team will be documented on the Delegation of Authority Form. The study coordinator and research assistants will be responsible for the informed consent process, review for eligibility, questionnaire administration, data entry, device administration, and e-CRF entries. The study coordinator will be responsible for AE/SAE documentation and reporting, while the investigator will be responsible for the AE assessment, review of the AE documentation forms, and overview of the research staff.

## 12.6. Protocol Deviations

All deviations from the protocol will be addressed in study participant source documents. The researcher will complete a Protocol Deviation Log using the participant code as the identifier. This form will collect information such as the date the deviation occurred, details of what the deviation consisted of, any corrective and preventative actions that were taken as a result of the deviation, and the date that the investigator and REB were notified. The investigator will review the information and initial once approved. A completed copy of the Protocol Deviation Form will be maintained in the regulatory file, as well as in the participant’s source document. Protocol deviations will be sent to the REB as per their guidelines. The site PI/study staff will be responsible for knowing and adhering to their REB requirements.

# 13. Ethical Considerations

## 13.1. Ethical Standard

The Principal Investigator will ensure that this study is conducted in full conformity with the ethical principles that have their origins in the Declaration of Helsinki. These principles are outlined by the Tri-Council Policy Statement: Ethical Conduct for Research Involving Humans - TCPS2 (2018, <https://ethics.gc.ca/eng/policy-politique_tcps2-eptc2_2018.html>) and codified in the International Council of Harmonization – Good Clinical Practice (ICH-GCP) E6.

## 13.2. Research Ethics Boards (REBs)

Research Ethics Boards of the participating sites must approve the protocol and informed consent document. The investigator will provide the Study Monitor/Coordinator with evidence that their REB has approved the study before participants are enrolled in the study at that site.

The study will be conducted in accordance with the protocol. The investigators will provide the REBs with information about any changes that are made to the protocol by submitting amendments. Other ongoing information will be submitted to the REBs including information on serious or unexpected adverse events.

## 13.3. Informed Consent Process

Informed consent is a process that is initiated prior to the individual’s agreement to participate in the study and continued throughout the individuals’ study participation. Extensive discussion of risks and possible benefits of FES will be provided to the participants.

1. Informed consent must be obtained prior to any study-specific procedure being conducted on the patient.
2. Obtaining personal written informed consent:
   a. The information is to be given to the subject in written format, the ICF
      i. Verify that the ICF given to the potential subject is the most recent REB-approved version of the consent for that clinical trial.
      ii. The ICF can be first presented to the patient by the Study Coordinator or other qualified personnel.
   b. The potential subject must be given ample time to read and understand the ICF.
   c. Study staff will then return and discuss the ICF with the potential patient, making sure to highlight important areas of the consent such as:
      1. The purpose of the study.
      2. Explain how many subjects will be involved.
      3. Discuss the duration of the study.
      4. Explain how many visits the subject will make.
      5. Discuss procedures; study intervention, device.
      6. Risks and possible benefits.
      7. Responsibilities of investigator and subject.
      8. Any compensation to the subject.
      9. Notification of any other treatment available for the condition.
      10. That the subject has the right to withdraw.
      11. Confidentiality.
      ii. Promote the exchange of information with the subject and encourage any questions.
   d. The Principal Investigator (or a Co-Investigator) should then join the potential subject and the subject is then given an additional opportunity to ask questions.
3. The ICF will then be signed by the subject and study staff presenting the ICF.
   a. The original ICF will be kept separate from any documentation containing the subject’s study code.
   b. A copy of the ICF will be given to the subject.

## 13.4. Exclusion of women, minorities, and children (special populations)

Non-English-speaking individuals are excluded as the ability to accurately and completely communicate study information, answer questions about the study, and obtain consent is necessary. Female participants will be asked if there is any reason to believe they might be pregnant. Pregnant participants will be excluded despite the fact that the theoretical risk to mother or fetus is exceedingly small since no safety data for pregnancy is known to exist for the FES. All women of childbearing potential will be asked to confirm that they are not pregnant, and they will immediately inform the study coordinator if they find out that they are pregnant.

## 13.5. Confidentiality

Participant confidentiality is strictly held in trust by the participating investigators, their staff, and the research team. This confidentiality is extended to cover testing of biological samples in addition to the clinical information relating to participants. All data will only be referenced by numerical identifier code. Data will be stored on a password-protected computer. A key connecting names and code numbers will be kept in a locked cabinet, accessible only by research personnel. All data will be stored and analyzed on password-protected computers, also only accessible by research personnel. Participants will not be identified in any report or publication about this study. See *12.2. Privacy, Security, and Data Confidentiality* for more information on source documentation storage and security.

## 13.6. Study Discontinuation

In the event that the study is discontinued, subjects who have completed or who are still enrolled in the study will be notified. Any new information gained during the course of the study that might affect subjects’ safety or willingness to continue participation in the study will be communicated to participants by the study coordinator within 2 days after the Principal Investigator learns this information.

# 14. Data Analysis

## 14.1. Sample Size Considerations

The sample size is based on our pilot study (17). In the pilot study, ten individuals with moderate to severe MDD received three FES sessions/week for a minimum of 10 to a maximum of 40 sessions. All study participants completed the required 10 therapy sessions, and 5 of the 10 participants decided to proceed with more sessions and they all completed additional 30 (total 40) FES sessions (17). This project has 20 days of FES intervention (once per day for 20 sessions) with a sham arm, so we anticipate a conservative attrition rate of 15% based on previous CAN-BIND trials with similar populations and neurostimulation literature. Accordingly, for this pilot trial, we aim to recruit 60 participants in total. A sample size of 30 per group will give us precise estimates of variability in the primary outcome, namely a change in HAM-D-17 score over time. Allowing for a conservative 15% overall drop-out rate, we will have roughly 51 participants. This number exceeds the recommended total sample sizes for a sufficiently precise estimate of the standard deviation and correlation for HAM-D-17 scores to use in the future sample size calculation for a definitive larger scale trial (between 24 and 50). With a total sample size of 60, we will be able to estimate a tolerability outcome, namely a drop-out rate of 15% to within a 95% confidence interval of ± 8.4%. Therefore, a sample size of 60 will be a conservative target within the limitations of the pilot study.

## 14.2. Outcomes Analysis

We are primarily interested in precise estimates of effectiveness and tolerability of FES compared to sham FES in patients with MDD, which will aid in the planning of a larger, sufficiently powered efficacy trial. Initial analyses will summarize socio-demographic and clinical characteristics by treatment group. Primary analyses will be performed on the modified intention-to-treat (ITT) population, including all randomized participants who received at least one FES treatment. The tolerability outcome, defined as overall drop-out rates, will be summarized using descriptive statistics such as counts and percentages. The efficacy analysis will report the between-group difference in change from baseline to Treatment 20 in the HAM-D-17 and its 95% confidence interval, using analysis of covariance (ANCOVA), with 20-treatment HAM-D-17 as the outcome, and baseline HAM-D-17 and stratification variables (sex, gender) as covariates. We will also report estimates for the standard deviation of HAM-D-17 score and within-person correlation between baseline and follow-up HAM-D-17 scores. A similar approach will be used for all continuous variables for secondary outcome measures (e.g., QIDS-SR, GAD-7, etc.). Differences between groups in dichotomous outcomes (e.g., response, remission, adverse effects) will be described using an odds ratio and a 95% confidence interval. To explore the sustainability of the treatment effect, we aim to also report changes in HAM-D-17 scores during the month after 20 treatments. The success of blinding patients will be assessed by computation of Bang’s blinding index. Sex and gender stratified analysis will be carried out.

Additionally, exploratory analyses of facial expressivity will be conducted using video data from the emotional recall interviews. Automated facial coding (OpenFace) will	quantify Duchenne smile metrics, including frequency, duration and average activation intensity. Descriptive statistics and group comparisons will be performed to examine changes in genuine smiling between FES and sham groups.

OpenFace is an open-source facial-behavior analysis program developed by academic researchers (University of Cambridge/Carnegie Mellon). It uses computer-vision and machine-learning algorithms to detect facial landmarks (e.g., eye, mouth, eyebrow positions), estimate head pose, and measure facial muscle activity (Action Units). In this study, OpenFace is not used for biometric facial recognition or identity matching. It is used only to convert each video into numerical measures of facial movement (e.g., amount of smiling over time).

The participant’s video is imported into OpenFace to extract Action Unit values and facial landmark data. The output is a CSV file containing numbers linked only to the participant’s study ID. These numeric files are what we will use for analysis.

# 15. Publication Policy and Dissemination

## 15.1. Clinical Trial Registration

This trial will be registered at www.clinicaltrials.gov in accordance with the policy from the International Committee of Medical Journal Editors^66^.

## 15.2. Declaration of Interests

The Principal Investigator of this trial, Dr. Venkat Bhat, has no financial or competing interests to declare.

Dr. Popovic has a patent approved February 16, 2016, with Dr. S. Hitzig and Dr. J Zariffa “Functional electrical stimulation method, use and apparatus for mood alteration”. The applicant is the University Health Network. Mood alteration is what we are doing in this study.

Dr. Zariffa is the patent inventor for functional electrical stimulation to modulate affect; the patent belongs to University Health Network; he has received research support from the Natural Sciences and Engineering Research Council of Canada, the Craig H. Neilsen Foundation, the Heart and Stroke Foundation of Canada, the Wings for Life Spinal Cord Research Foundation, the Ontario Ministry of Colleges and Universities, and the Praxis Spinal Cord Institute. Dr. Zariffa has also received research support from the Canadian Institutes of Health Research (CIHR).

## 15.3. Dissemination Policy

The results of this trial will be presented at scientific conferences and published, using the CONSORT guidelines, in peer-reviewed scientific journals.

## 15.4. Authorship

To be eligible for authorship on any resultant publications, all potential contributors must fulfill all criteria as set forth by the International Committee of Medical Journal Editors.

# 16. References

1.	Sheehan DV, Lecrubier Y, Sheehan KH, et al. The Mini-International Neuropsychiatric Interview (MINI): the development and validation of a structured diagnostic psychiatric interview for DSM-IV and ICD-10. The Journal of clinical psychiatry 1998.

2.	Milev RV, Giacobbe P, Kennedy SH, et al. Canadian Network for Mood and Anxiety Treatments (CANMAT) 2016 clinical guidelines for the management of adults with major depressive disorder: section 4. Neurostimulation treatments. The Canadian Journal of Psychiatry 2016;61:561-75.

3.	Berman RM, Narasimhan M, Charney DS. Treatment‐refractory depression: definitions and characteristics. Depression and anxiety 1997;5:154-64.

4.	Health Quality Ontario. Repetitive transcranial magnetic stimulation for treatment-resistant depression: a systematic review and meta-analysis of randomized controlled trials. Ontario health technology assessment series 2016;16:1.

5.	Martin-Vazquez M-J. Adherence to antidepressants: a review of the literature. Neuropsychiatry 2016;6:236–41.

6.	Bayes A, Parker G. Comparison of guidelines for the treatment of unipolar depression: a focus on pharmacotherapy and neurostimulation. Acta Psychiatrica Scandinavica 2018;137:459-71.

7.	Petrides G, Dhossche D, Fink M, Francis A. Continuation ECT: Relapse prevention in affective disorders. Convulsive therapy 1994.

8.	Rachid F. Maintenance repetitive transcranial magnetic stimulation (rTMS) for relapse prevention in with depression: a review. Psychiatry research 2018;262:363-72.

9.	Meron D, Hedger N, Garner M, Baldwin DS. Transcranial direct current stimulation (tDCS) in the treatment of depression: systematic review and meta-analysis of efficacy and tolerability. Neuroscience & Biobehavioral Reviews 2015;57:46-62.

10.	Richards D, Richardson T. Computer-based psychological treatments for depression: a systematic review and meta-analysis. Clinical psychology review 2012;32:329-42.

11.	Al-Karawi D, Jubair L. Bright light therapy for nonseasonal depression: meta-analysis of clinical trials. Journal of affective disorders 2016;198:64-71.

12.	Elias GJB, Boutet A, Parmar R, et al. Neuromodulatory treatments for psychiatric disease: A comprehensive survey of the clinical trial landscape. Brain Stimul 2021;14:1393-403.

13.	Demchenko I BD, Flint A, Anderson M, Daskalakis Z, Foley K, Karkouti K, Kennedy SH, Ladha, K, Robertson J, Vaisman A, Koczerginski D, Parikh SV, Bhat V. Electroconvulsive Therapy in Canada During the First Wave of COVID-19: Results of The “What Happened” National Survey. Journal of ECT 2021, In Press.

14.	Tomkins SS. Affect imagery consciousness: Volume I: The positive affects: Springer publishing company; 1962.

15.	Zariffa J, Hitzig SL, Popovic MR. Neuromodulation of emotion using functional electrical stimulation applied to facial muscles. Neuromodulation: technology at the neural interface 2014;17:85-92.

16.	Zariffa J, Hitzig SL, Popovic MR. Functional Electrical Stimulation Applied to Facial Muscles can be used to Neuromodulate Emotions.

17.	Kapadia N, Zivanovic V, Moineau B, Downar J, Zariffa J, Popovic MR. Functional electrical stimulation of the facial muscles to improve symptoms in individuals with major depressive disorder: pilot feasibility study. Biomedical engineering online 2019;18:1-17.

18.	Iwasa S DI, Kapadia-Desai N, Zariffa J, Mulsant B, Kennedy SH, Popovic M, Bhat V. . Stimulating Facial Musculature as a Therapeutic Intervention for Depression: A Conceptual Overview. Frontiers in Psychiatry 2021, Revisions requested;7:94-108.

19.	Demchenko I, Kapadia-Desai N, Iwasa S, et al. Botulinum Toxin for Depression: A Systematic Review of the Clinical Research Landscape. Journal of Affective Disorders 2021, Revisions requested;6:400-22.

20.	Hebert DA, Bowen JM, Ho C, Antunes I, O’Reilly DJ, Bayley M. Examining a new functional electrical stimulation therapy with people with severe upper extremity hemiparesis and chronic stroke: a feasibility study. British journal of occupational therapy 2017;80:651-9.

21.	Murray CJ, Lopez AD, Organization WH. The global burden of disease: a comprehensive assessment of mortality and disability from diseases, injuries, and risk factors in 1990 and projected to 2020: summary: World Health Organization; 1996.

22.	Gartlehner G, Hansen RA, Morgan LC, et al. Second-generation antidepressants in the pharmacologic treatment of adult depression: an update of the 2007 comparative effectiveness review [Internet]. 2011.

23.	Gartlehner G, Gaynes BN, Amick HR, et al. Nonpharmacological versus pharmacological treatments for adult patients with major depressive disorder. 2015.

24.	Mutz J, Vipulananthan V, Carter B, Hurlemann R, Fu CH, Young AH. Comparative efficacy and acceptability of non-surgical brain stimulation for the acute treatment of adult major depressive episodes: A systematic review and network meta-analysis of 113 randomised clinical trials. bioRxiv 2018:426866.

25.	Seth AK, Friston KJ. Active interoceptive inference and the emotional brain. Philosophical Transactions of the Royal Society B: Biological Sciences 2016;371:20160007.

26.	Bhat V, Kennedy SH. Vagus Nerve Stimulation: A Treatment in Evolution. Cogn Behav Neurol 2018;31:99-100.

27.	Conway CR, Xiong W. The mechanism of action of vagus nerve stimulation in treatment-resistant depression: current conceptualizations. Psychiatric Clinics 2018;41:395-407.

28.	Parsaik AK, Mascarenhas SS, Hashmi A, et al. Role of botulinum toxin in depression. Journal of Psychiatric Practice® 2016;22:99-110.

29.	Popovic MR, Kapadia N, Zivanovic V, Furlan JC, Craven BC, McGillivray C. Functional electrical stimulation therapy of voluntary grasping versus only conventional rehabilitation for patients with subacute incomplete tetraplegia: a randomized clinical trial. Neurorehabilitation and neural repair 2011;25:433-42.

30.	Howlett OA, Lannin NA, Ada L, McKinstry C. Functional electrical stimulation improves activity after stroke: a systematic review with meta-analysis. Archives of physical medicine and rehabilitation 2015;96:934-43.

31.	Beaumont E, Guevara E, Dubeau S, Lesage F, Nagai M, Popovic M. Functional electrical stimulation post-spinal cord injury improves locomotion and increases afferent input into the central nervous system in rats. The journal of spinal cord medicine 2014;37:93-100.

32.	Schwanenberg E. Izard, CE: The Face of Emotion. New York (Appleton-Century-Crofts) 1971, 468 Seiten. Psyche 1974;28:919-20.

33.	Tourangeau R, Ellsworth PC. The role of facial response in the experience of emotion. Journal of Personality and Social Psychology 1979;37:1519.

34.	Ekman P, Davidson RJ, Friesen WV. The Duchenne smile: emotional expression and brain physiology: II. Journal of personality and social psychology 1990;58:342.

35.	Parr LA, Waller BM, Vick SJ, Bard KA. Classifying chimpanzee facial expressions using muscle action. Emotion 2007;7:172.

36.	Ekman P, Davidson RJ. Voluntary smiling changes regional brain activity. Psychological Science 1993;4:342-5.

37.	Wiswede D, Münte TF, Krämer UM, Rüsseler J. Embodied emotion modulates neural signature of performance monitoring. PLoS One 2009;4:e5754.

38.	Frank MG, Ekman P. Physiologic effects of the smile. Directions in Psychiatry 1996;16:1-8.

39.	Soussignan R. Duchenne smile, emotional experience, and autonomic reactivity: a test of the facial feedback hypothesis. Emotion 2002;2:52.

40.	Bell ML, Kenward MG, Fairclough DL, Horton NJ. Differential dropout and bias in randomised controlled trials: when it matters and when it may not. Bmj 2013;346.

41.	Duecker F, Sack AT. Rethinking the role of sham TMS. Frontiers in psychology 2015;6:210.

42.	Montgomery SA, Åsberg M. A new depression scale designed to be sensitive to change. The British Journal of Psychiatry 1979;134:382-9.

43.	Grover S, Sahoo S, Dua D, Chakrabarti S, Avasthi A. Scales for assessment of depression in schizophrenia: Factor analysis of calgary depression rating scale and hamilton depression rating scale. Psychiatry Res 2017;252:333-9.

44.	Surís A, Holder N, Holliday R, Clem M. Psychometric validation of the 16 Item Quick Inventory of Depressive Symptomatology Self-Report Version (QIDS-SR16) in military veterans with PTSD. J Affect Disord 2016;202:16-22.

45.	Spitzer RL, Kroenke K, Williams JB, Lowe B. A brief measure for assessing generalized anxiety disorder: the GAD-7. Arch Intern Med 2006;166:1092-7.

46.	Mortazavi F, Mousavi SA, Chaman R, Khosravi A. [Validation of the World Health Organization-5 Well-Being Index; assessment of maternal well-being and its associated factors]. Turk Psikiyatri Derg 2015;26:48-55.

47.	Buysse DJ, Reynolds CF, 3rd, Monk TH, Berman SR, Kupfer DJ. The Pittsburgh Sleep Quality Index: a new instrument for psychiatric practice and research. Psychiatry Res 1989;28:193-213.

48.	Doig GS, Simpson F. Randomization and allocation concealment: a practical guide for researchers. Journal of critical care 2005;20:187-91.

49.	Kolahi J, Bang H, Park J. Towards a proposal for assessment of blinding success in clinical trials: up‐to‐date review. Community dentistry and oral epidemiology 2009;37:477-84.

50.	Ekman P, Friesen WV. Facial action coding system: Investigator's guide: Consulting Psychologists Press; 1978.

51.	Grasin E, Loginov I, Masliukova A, Smirnov N. Realistic sham TMS. Brain Stimulation: Basic, Translational, and Clinical Research in Neuromodulation 2019;12:418.

52.	Bikson M, Brunoni AR, Charvet LE, et al. Rigor and reproducibility in research with transcranial electrical stimulation: an NIMH-sponsored workshop. Brain stimulation 2018;11:465-80.

53.	Baer L, Blais MA. Handbook of clinical rating scales and assessment in psychiatry and mental health: Springer; 2010.

54.	Hamilton M. A rating scale for depression. Journal of neurology, neurosurgery, and psychiatry 1960;23:56.

55.	Rush AJ, Trivedi MH, Ibrahim HM, et al. The 16-Item Quick Inventory of Depressive Symptomatology (QIDS), clinician rating (QIDS-C), and self-report (QIDS-SR): a psychometric evaluation in patients with chronic major depression. Biological psychiatry 2003;54:573-83.

56.	Spitzer RL, Kroenke K, Williams JB, Löwe B. A brief measure for assessing generalized anxiety disorder: the GAD-7. Archives of internal medicine 2006;166:1092-7.

57.	Lex H, Ginsburg Y, Sitzmann AF, Grayhack C, Maixner DF, Mickey BJ. Quality of life across domains among individuals with treatment-resistant depression. Journal of affective disorders 2019;243:401-7.

58.	Brunelin J, Mondino M, Gassab L, et al. Examining Transcranial Direct-Current Stimulation (tDCS) as a Treatment for Hallucinations in Schizophrenia. American Journal of Psychiatry 2012;169:719-24.

59.	Berlim MT, Van den Eynde F, Daskalakis ZJ. Clinical utility of transcranial direct current stimulation (tDCS) for treating major depression: a systematic review and meta-analysis of randomized, double-blind and sham-controlled trials. J Psychiatr Res 2013;47:1-7.

60.	Korn EL, Freidlin B, Abrams JS, Halabi S. Design issues in randomized phase II/III trials. Journal of Clinical Oncology 2012;30:667.

61.	Cohen J. Statistical power analysis for the behavioral sciences: Academic press; 2013.

62.	Billingham SA, Whitehead AL, Julious SA. An audit of sample sizes for pilot and feasibility trials being undertaken in the United Kingdom registered in the United Kingdom Clinical Research Network database. BMC Med Res Methodol 2013;13:104.

63.	Sim J, Lewis M. The size of a pilot study for a clinical trial should be calculated in relation to considerations of precision and efficiency. J Clin Epidemiol 2012;65:301-8.

64.	Julious SA. Sample size of 12 per group rule of thumb for a pilot study. Pharm Stat J Appl Stat Pharm Ind 2005;4:287-91.

65.	Bang H, Flaherty SP, Kolahi J, Park J. Blinding assessment in clinical trials: a review of statistical methods and a proposal of blinding assessment protocol. Clinical Research and Regulatory Affairs 2010;27:42-51.

66.	De Angelis CD, Drazen JM, Frizelle FA, et al. Is this clinical trial fully registered?--A statement from the International Committee of Medical Journal Editors. N Engl J Med 2005;352:2436-8.

67. American Psychiatric Association. Diagnostic and statistical manual of mental disorders, Fifth Edition. Arlington, VA: Americal Psychiatric Publishing, Inc.; 2013.

68.	Folstein MF, Folstein SE, McHugh PR. "Mini-mental state". A practical method for grading the cognitive state of patients for the clinician. J Psychiatr Res. 1975;12(3):189-98.

69.	Oquendo MA, Baca-Garcia E, Kartachov A, Khait V, Campbell CE, Richards M, et al. A computer algorithm for calculating the adequacy of antidepressant treatment in unipolar and bipolar depression. J Clin Psychiatry. 2003;64(7):825-33.

70.	Sackeim HA, Prudic J, Devanand DP, Decina P, Kerr B, Malitz S. The impact of medication resistance and continuation pharmacotherapy on relapse following response to electroconvulsive therapy in major depression. J Clin Psychopharmacol. 1990;10(2):96-104.

71.	Montgomery SA, Åsberg M. A new depression scale designed to be sensitive to change. The British Journal of Psychiatry. 1979;134:382-9.

81. Kennedy SH, Lam RW, Rotzinger S, et al. Symptomatic and functional outcomes and early prediction of response to escitalopram monotherapy and sequential adjunctive aripiprazole therapy in patients with major depressive disorder: a CAN-BIND-1 report. The Journal of clinical psychiatry 2019;80:0-.

82. Berlim M, Van den Eynde F, Tovar-Perdomo S, Daskalakis Z. Response, remission and drop-out rates following high-frequency repetitive transcranial magnetic stimulation (rTMS) for treating major depression: a systematic review and meta-analysis of randomized, double-blind and sham-controlled trials. Psychological medicine 2014;44:225.
